# Engineering of Optoelectronic Devices for Renewable Energy Applications

**DOI:** 10.3390/mi17060758

**Published:** 2026-06-22

**Authors:** José Pereira, Reinaldo Souza, Ana Moita

**Affiliations:** 1 1 IN+ Center for Innovation, Technology and Policy Research, Instituto Superior Técnico, Universidade de Lisboa, Avenida Rovisco Pais, 1049-001 Lisboa, Portugal; reinaldo.souza@tecnico.ulisboa.pt (R.S.); anamoita@tecnico.ulisboa.pt (A.M.); 2 CINAMIL—Military Academy Research Center, Department of Exact Sciences and Engineering, Portuguese Military Academy, 2720-113 Amadora, Portugal

**Keywords:** optoelectronic devices, plasmonic materials, solar energy harvesting, photovoltaics, machine learning

## Abstract

Optoelectronic devices are emerging as a cornerstone of advanced renewable energy technologies, offering innovative routes for energy harvesting, conversion, and management with high efficiency and versatility. This review summarizes recent advances in the semiconductor materials engineering field, device configurations, and light–matter interaction mechanisms that underpin advanced optoelectronic systems for solar energy harvesting, solar-driven chemical conversion, and smart grid integration, among others. Emphasis is placed on the breakthroughs achieved in the perovskite and hybrid photovoltaics, photoelectrochemical energy conversion, and nanostructured optoelectronic platforms that enable much-increased light absorption, reduced recombination losses, and scalable large-scale fabrications. Moreover, the challenges closely linked with long-term stability, environmental durability and benevolence, and worldwide deployment are critically addressed, together with the emerging opportunities in AI design, tandem device technological solutions, integrated energy systems, and machine learning approaches for optimizing device performance, thermal management, and energy storage capabilities. Finally, the present review concludes by outlining the future research directions that could accelerate the transition toward high-performance, cost-effective, and sustainable optoelectronic solutions responsive to global renewable energy requirements.

## 1. Introduction

The accelerating demand for clean, reliable, and scalable energy solutions has placed renewable technologies at the forefront of global scientific and industrial research. As nations work to decarbonize their economies and transition away from fossil fuel dependence, the need for high-efficiency energy harvesting, conversion, and management technologies has never been more urgent. Within this landscape, optoelectronic devices—systems that exploit light–matter interactions in electronic materials—have emerged as key enablers of next-generation renewable energy technologies. Their ability to manipulate photons and charge carriers with precision makes them indispensable in applications such as solar photovoltaics, photoelectrochemical fuel generation, optical sensing, and smart-grid communication.

Optoelectronics has undergone a profound transformation over the past two decades. Traditional semiconductor platforms such as crystalline silicon and III–V compounds have been complemented—and in some cases challenged—by a new generation of materials, including metal-halide perovskites, organic semiconductors, quantum dots, and two-dimensional transition-metal dichalcogenides [1]. These emerging materials offer tunable bandgaps [2], low-temperature processing, mechanical flexibility, and compatibility with large-area manufacturing [3]. In parallel, the progress in nanophotonics, plasmonics, and nano-optics [4] has enabled refined control over light absorption, scattering, and confinement, bringing device efficiencies closer to their theoretical limits.

The relevance of optoelectronic engineering extends far beyond electricity generation. Solar-driven chemical processes—including photoelectrochemical water splitting [5], CO_2_ reduction to synthetic fuels, and photocatalytic pollutant degradation [6]—rely on advanced light-responsive materials and interfaces. Meanwhile, the integration of optical sensors, photodetectors, and communication modules into renewable energy infrastructures is redefining how grids monitor performance, predict failures, and balance supply and demand. These developments highlight a broader trend as renewable energy systems are becoming increasingly multifunctional, intelligent, and interconnected, with optoelectronic materials and devices serving as an integrated technology base.

Despite the remarkable progress, some significant challenges remain. Many high-performance materials suffer from instability under heat, moisture, and prolonged illumination. Scalable manufacturing routes must balance investment cost, environmental impact, and long-term reliability. Device architectures must be optimized not only for peak efficiency but also for durability, recyclability, and integration with storage and power-management systems. Addressing these challenges requires an integrated understanding of materials science, device physics, and engineering design [7].

To provide a coherent and transparent structure, this manuscript focuses in depth on the fundamental materials, device architectures, and manufacturing strategies that underpin optoelectronic renewable-energy technologies. Sections on photovoltaics, solar-fuel generation, and optoelectronic sensing are treated comprehensively, with detailed discussions of material properties, device physics, and performance-limiting mechanisms. In contrast, system-level topics—such as grid integration, life cycle assessment, and policy frameworks—are included to contextualize the technological landscape and highlight cross-cutting constraints but are not intended as exhaustive surveys. This organizational strategy ensures that the review remains technically rigorous while situating device-level advances within the broader renewable-energy ecosystem.

By connecting developments across photovoltaics, solar fuels, optical sensing, and grid technologies, this review underscores the central role of optoelectronics in shaping a sustainable energy future and outlines the main research directions that could expedite progress in the coming decades.

## 2. Fundamentals of Optoelectronic Devices

The optoelectronic devices operate at the intersection of photonics and electronics, relying on the ability of materials to absorb, emit, and modulate light while simultaneously transporting electrical charge [8]. Their operation is governed by a combination of semiconductor physics, light–matter interaction principles, and device architectures that optimize charge carrier generation, separation, and collection. These principles provide the foundation for understanding how optoelectronic systems enable high-performance renewable energy technologies.

### 2.1. Light–Matter Interaction in Semiconductors

When a semiconductor absorbs a photon with energy greater than its bandgap, it generates an electron–hole pair or an exciton. The efficiency of this process depends on the absorption coefficient, band structure, and material dimensionality. Charge carriers can recombine radiatively (via photon emission) or through defect-mediated non-radiative pathways [9]. Minimizing non-radiative recombination losses is essential for high-efficiency solar cells [10] and light-driven catalytic systems. Once generated, charge carriers must travel through the material without recombining, and their mobility, diffusion length [11], and scattering mechanisms [12] determine how effectively they reach device contacts and reactive interfaces. The bandgap determines which wavelengths a material can absorb. Techniques such as quantum confinement [13] and bandgap engineering [2] allow precise control over optical and electronic properties. Direct bandgap materials, such as gallium arsenide and perovskites [14], absorb light efficiently, whereas indirect bandgap materials, such as silicon, require thicker layers and advanced light-management strategies. Crystal lattice imperfections introduce energy levels that can trap charge carriers, thereby reducing device performance [15]. Finally, passivation strategies are crucial for improving device stability and efficiency [16].

### 2.2. Device Configurations and Metrics

The p–n junctions and heterojunctions create internal electric fields that separate photogenerated carriers, while heterojunctions enable band alignment engineering for improved charge extraction [17]. In Schottky barriers and metal–semiconductor interfaces, metal contacts form barriers that influence carrier injection and extraction [18]. In addition, thin-film and nanostructured materials [19], such as nanowires, quantum dots, and textured surfaces, enhance light absorption through trapping effects and increase the surface area available for catalytic reactions. Antireflection coatings minimize reflection losses and increase light coupling into the device [20]. Plasmonic and photonic structures, such as metallic nanoparticles, metasurfaces, and photonic crystals, concentrate light at the nanoscale, considerably increasing absorption in the thin active layers. Furthermore, textured substrates and scattering layers extend the optical path length, enhancing light absorption efficiency even in ultrathin devices. Selective contacts, including electron- and hole-selective layers, ensure unidirectional carrier flow, reducing the recombination at the interfaces [21]. Surface passivation treatments and thin dielectric layers suppress surface defects that act as recombination centers. Proper alignment between the absorber materials and the transport layers minimizes the energy barriers and enhances power conversion efficiency and open-circuit voltage [22]. In terms of fundamental metrics, the external and internal quantum efficiencies quantify how absorbed photons generate usable charge. The open-circuit voltage and the fill factor reflect recombination losses and resistive effects within the device. The Shockley–Queisser limit and related models define the theoretical maximum efficiency for single-junction and tandem devices [23].

## 3. Materials for Optoelectronic Renewable Energy Devices

### 3.1. Semiconductors

Traditional semiconductor materials form the technological backbone of modern optoelectronic and renewable energy devices. Their manufacturing methods, theoretical understanding, and proven long-term reliability make them indispensable benchmarks against which emerging materials are compared. Silicon, gallium arsenide, and indium phosphide offer distinct advantages arising from their electronic structure, optical properties, and ease of manufacturing. Silicon remains the dominant material in photovoltaic (PV) technology, accounting for more than 90% of the global solar cell market [24]. Its success stems from a combination of abundance, non-toxicity, and compatibility with large-scale manufacturing. Abundant and environmentally benign, silicon is derived from silica, making it one of the most common elements and a sustainable option for large-scale deployment. The indirect bandgap of silicon (~1.12 eV), although requiring thicker absorber layers to achieve high light absorption, also contributes to excellent carrier lifetimes and stability [25]. The long diffusion lengths of silicon, together with advances in crystal growth and wafer processing, enable efficient charge transport and low recombination losses.

Additionally, silicon supports heterojunctions, passivated contacts, and tandem configurations with perovskites, enabling overall efficiencies of approximately 27% [26]. Gallium arsenide (GaAs) is a direct-bandgap III–V semiconductor recognized for its superior optoelectronic performance [27]. Although expensive, silicon delivers very high efficiencies and is used in high-value applications. Its direct bandgap (≈1.42 eV) enables high absorption and high open-circuit voltages, making GaAs highly suitable for thin and lightweight solar cells [28]. GaAs also maintains performance under extreme radiation conditions, which is why it dominates space-grade photovoltaics. Its high electron mobility facilitates fast carrier transport, supporting high-frequency optoelectronic devices and photodetectors [29]. It is also compatible with multijunction cells, as GaAs serves as a key component in III–V tandem and triple-junction configurations exceeding 40% efficiency [30].

Indium phosphide (InP) is another high-performance III–V semiconductor with advantages for photovoltaics and photoelectrochemical systems. The direct bandgap of InP around 1.34 eV matches the solar spectrum, enabling effective photon absorption and high photovoltage [31]. The surface passivation of InP can be further improved using elements such as sulfur [32], sodium sulfide [33], hexagonal boron nitride [34], and techniques involving the removal of the multiple inherent dangling bonds [35] of InP. It displays high electron velocity and mobility, supporting fast optoelectronic devices, including high-speed photodetectors and communication components. The behavior of InP in aqueous environments makes it a suitable photoanode for solar-driven hydrogen evolution [36] and for photoelectrochemical reactions in pollutant removal procedures [37]. Although silicon, GaAs, and InP differ in cost, processing ease, and performance, these materials define the fundamental basis of optoelectronic systems. Table 1 presents the characteristics, benefits and applications of these materials.

### 3.2. Perovskites, Organic Semiconductors, and Transition-Metal Dichalcogenides

The emerging optoelectronic materials have reshaped the landscape of renewable energy research by offering tunable properties, low-temperature processing, and compatibility with flexible and lightweight devices. Among these, metal-halide perovskites, organic semiconductors, and two-dimensional transition-metal dichalcogenides (TMDs) stand out due to their rapid performance improvements and distinctive physical properties. Each class offers advantages that complement, and in some cases surpass, traditional semiconductors, making them highly relevant for advanced solar cells, photodetectors, and solar fuel systems.

Metal-halide perovskites are solution-processable semiconductors [38] with the general ABX_3_ crystal structure, where a monovalent cation (A), a divalent metal (B), and a halide anion (X) form a corner-sharing octahedral framework. This lattice is highly tolerant to substitution, which enables bandgap tuning across roughly 1.2–2.3 eV through halide mixing (Cl/Br/I) and cation engineering. As a result, perovskites can be tailored for single-junction absorbers, wide-bandgap tandem top cells, and narrow-bandgap bottom cells [39]. Their fundamental optoelectronic strengths include a high absorption coefficient [40], long carrier diffusion lengths and lifetimes [41], and low defect densities, allowing efficient charge generation in ultrathin films [42]. These properties, together with low-temperature deposition methods such as spin-coating [43], blade coating [44], vapor deposition [45], and inkjet printing [46], make perovskites uniquely attractive for next-generation optoelectronic devices. The main challenges associated with perovskites are instability under moisture [47], elevated temperatures [48], UV exposure [49], ion migration [50], and lead toxicity and leakage [51], which remain barriers to broad commercialization. A detailed discussion of their photovoltaic operation, device configurations, and performance limits is provided in Section 4.2.

On the other hand, organic semiconductors, which are based on π-conjugated polymers [52] and small molecules, offer mechanical flexibility, lightweight construction, and compatibility with roll-to-roll manufacturing. These materials enable low-temperature solution processing, allowing large-area fabrication on polymeric substrates, which is suitable for portable and wearable solar technologies. In addition, organic semiconductors have tunable molecular structures, as their chemical synthesis allows control over bandgap, energy levels, and charge transport properties. The exciton binding energies in the range of 0.3–1 eV require engineered donor–acceptor interfaces for efficient charge separation [53]. The primary application fields are organic photovoltaics (OPVs) [54], flexible photodetectors [55], and semitransparent solar cells [56]. The main challenges are related to limited operational stability and lower carrier mobility when compared to inorganic materials, and degradation under oxygen and light [57]. Moreover, transition-metal dichalcogenides (TMDs) such as MoS_2_, WS_2_, and MoSe_2_ are atomically thin semiconductors exhibiting strong light–matter interactions and improved electronic properties. Many TMDs transition from indirect to direct bandgaps when thinned to a monolayer, enhancing absorption and emission capabilities. They exhibit excellent mechanical flexibility, enabling ultrathin and bendable optoelectronic devices and integration with unconventional substrates [58].

The strong Coulomb interactions further enhance light absorption [59] and excitonic effects. The main applications of TMDs are photodetectors, flexible solar cells, catalytic interfaces for hydrogen evolution [60,61], and components for tandem and hybrid equipment [62]. However, challenges remain in scalable synthesis by Chemical Vapor Deposition (CVD), exfoliation, and restacking processes [63]. Despite their remarkable performance, these emerging materials present trade-offs in terms of stability, scalability, and environmental impact. Perovskites achieve the best efficiencies but suffer from instability and toxicity concerns, while organic semiconductors provide flexibility but are limited by lower carrier mobility. TMDs exhibit unique electronic properties but face challenges in large-scale fabrication. Table 2 summarizes the main benefits and challenges of these material classes (perovskites, organic semiconductors, and TMDs).

### 3.3. Nanostructured Materials

Nanostructured materials have opened new pathways for modern optoelectronic devices with enhanced light absorption, tailored electronic properties, and improved charge carrier dynamics [64]. Their unique quantum-scale behavior enables functionalities that are difficult or impossible to achieve with bulk semiconductors. In renewable energy technologies, nanostructuring plays a fundamental role in enhancing efficiency, reducing material consumption, and enabling flexible device configurations. Quantum dots (QDs) are semiconductor nanocrystals whose electronic and optical properties are governed by quantum confinement [65]. Their size-dependent bandgap and strong absorption make them highly promising for solar energy conversion and photocatalytic applications [66]. Adjusting the QD diameter enables precise control over absorption and emission wavelengths, supporting concepts such as multijunction and spectrum-splitting solar cells [67]. QDs can also exhibit Multiple Exciton Generation (MEG) [68], producing more than one electron–hole pair per absorbed high-energy photon and offering a pathway to surpass conventional photovoltaic efficiency limits. Colloidal QDs are solution-processable [69] and can be deposited via spin coating, printing, and spray coating, allowing for cost-effective, large-area fabrication. The main applications include QD-sensitized solar cells, hybrid perovskite–QD tandem devices, photocatalytic hydrogen evolution, and infrared photodetectors. Nevertheless, challenges such as surface trap states, ligand instability, and toxicity concerns, particularly for lead- and cadmium-based QDs [70], remain significant barriers to their widespread deployment. Moreover, semiconductor nanowires provide one-dimensional pathways for charge transport and exhibit enhanced light–matter interaction due to their high aspect ratio and waveguiding properties [71].

Radial p–n junctions in nanowires enable efficient charge separation and collection by shortening carrier diffusion distances and reducing recombination losses [72]. Silicon nanowire arrays also act as natural light-trapping structures, allowing strong optical absorption with minimal material thickness [73]. Their ability to relax strain makes them more tolerant to lattice mismatch than bulk films, facilitating the integration of III–V materials on silicon. Their applications include high-efficiency nanowire solar cells, photoelectrochemical water-splitting electrodes, and fast photodetectors [74]. Nonetheless, challenges such as complex growth processes (VLS, MOCVD), alignment control, and scalability remain significant barriers. Finally, plasmonic materials, mainly noble metal nanostructures such as gold and silver nanoparticles, and bimetallic and multi-metallic nanostructures [75] take advantage of the oscillations of conduction electrons to manipulate light at subwavelength scales.

The Localized Surface Plasmon Resonance (LSPR) enables strong near-field enhancement, significantly enhancing absorption in adjacent semiconductors [76]. The decay of plasmons can generate energetic hot-carriers capable of driving photocatalytic reactions and contributing to photocurrent. Metal nanostructures also increase light scattering and trapping by coupling incident photons into guided modes, increasing the optical path length in thin-film devices. The main applications include plasmon-enhanced solar cells [77], photocatalysis, Surface-Enhanced Raman Spectroscopy (SERS) biosensors [78], and hybrid photonic and plasmonic waveguides to enhance optical communication [79]. Despite these advantages, challenges such as optical losses in metals, thermal instability, and integration complexity continue to limit widespread adoption.

Overall, nanostructured materials enable significant performance enhancements but introduce new challenges in stability, scalability, and integration. Their combination with emerging semiconductors such as perovskites and TMDs presents a highly promising route for next-generation hybrid optoelectronic systems. Table 3 shows the fundamental benefits and challenges of nanostructured materials for optoelectronics.

The nanostructured materials continue to reshape the design of optoelectronic renewable energy devices, enabling configurations combining high efficiency with mechanical flexibility and low material consumption. Their integration with perovskites, organics, and TMDs is driving out some of the most promising hybrid systems in the current research.

### 3.4. Scalable Fabrication Methods for Nanostructured Optoelectronic Platforms

While nanostructured materials offer significant performance advantages in light management and charge dynamics, their potential industrial-scale production remains challenging due to issues related to uniformity, cost, and integration. The following methods are foreseen to be the most promising for practical large-scale manufacturing:

- Aerosol-liquid-solid (ALS) spraying, which enables the patterning of high-quality perovskite thick films with controllable bandgap and thickness on large substrates. It combines simplicity, scalability, and improved film quality, making it very suitable for multispectral and hybrid devices [80].
- Blade coating and slot-die coating, which are roll-to-roll compatible methods that allow a uniform deposition of nanostructured thin films and hybrid perovskites over large areas at high throughput and low cost [81].
- Microdroplet interface synthesis, which is a recently demonstrated methodology for growing oriented single crystals, such as lead-free double perovskites, with controlled facets. This method offers enhanced reproducibility and can be adapted for batch and continuous production [82].
- Inkjet printing and nanoimprint lithography, which are techniques that provide high-resolution patterning of quantum dots and plasmonic nanostructures while maintaining compatibility with flexible substrates [81].

Recent works have also demonstrated wafer-scale integration of highly oriented 2D perovskite oxide nanosheets by charge-assisted assembly, enabling ultra-flexible high-resolution photodetector arrays [83]. Despite these advances, some challenges for fully industrial adoption still remain:

- Achieving uniformity and defect control over meter-scale areas.
- Integration with existing silicon manufacturing infrastructure.
- Long-term stability of nanostructures under real operating conditions (moisture, thermal cycling, and light exposure).
- Cost-effective encapsulation and recycling processes for hybrid nanostructured devices.

Addressing these challenges through hybrid processing routes (combining solution-based methods with vapor deposition) and machine learning optimization will be critical for the widespread commercialization of nanostructured optoelectronic devices in renewable energy applications.

### 3.5. Stability, Toxicity, and Sustainability

The long-term viability of emerging optoelectronic materials depends on their intrinsic stability, toxicity profile, and sustainability. At the material level, different classes exhibit distinct degradation pathways driven by thermal, photochemical, and moisture-induced processes. Understanding these mechanisms is essential for selecting compositions capable of withstanding real-world environmental stressors.

Halide perovskites are susceptible to thermal instability and photochemical degradation, where elevated temperatures and prolonged illumination promote ion migration [84], lattice distortion, and halide segregation. These processes can induce phase transitions [85], vacancy formation, and bond cleavage within the soft ionic lattice. Additionally, sensitivity to moisture is another defying issue: water molecules readily penetrate perovskite films, promoting hydrolysis, dissolution of ionic species, and irreversible structural decomposition. Also, mixed-cation and mixed-halide formulations improve lattice rigidity and reduce phase instability [86], but susceptibility to ion migration remains a central limitation.

Organic semiconductors exhibit degradation pathways dominated by photo-oxidation and thermal bond scission. Their π-conjugated backbones and side-chain chemistries are vulnerable to reactions with oxygen and moisture under sunlight, leading to radical formation, chain cleavage, and loss of conjugation. Elevated temperatures accelerate these reactions and can also induce morphological rearrangements such as crystallite melting and phase separation. Although molecular design and green-solvent processing have reduced some of the environmental hazards, chemical fragility remains a fundamental constraint for long-term stability [86].

Transition-metal dichalcogenides (TMDs) generally possess stronger covalent bonding and higher thermal robustness, yet they are not immune to degradation [87]. Moisture-assisted oxidation can occur at defect sites or grain boundaries, forming oxides that alter electronic structure and carrier mobility. Also, photochemical reactions under high-energy illumination may also generate chalcogen vacancies and induce surface reconstruction. These effects are more pronounced in ultrathin and monolayer TMDs, where high surface-to-volume ratios amplify environmental reactivity.

Nanostructured materials, such as plasmonic nanoparticles, quantum dots, and colloidal nanocrystals, exhibit degradation pathways strongly influenced by their high surface area and ligand chemistry. Thermal exposure can drive ligand desorption, surface atom diffusion, and coalescence, while photochemical processes may induce oxidation or ion exchange at the surface. Furthermore, moisture accelerates these reactions and can destabilize the surface passivation layers, leading to aggregation or dissolution. These behaviors raise additional concerns regarding the toxicity of nanomaterials, as unbound nanoparticles may pose inhalation and bioaccumulation risks if released into the environment [88].

Toxicity considerations are central to the environmental safety of emerging materials. Lead-based perovskites offer exceptional optoelectronic performance but raise concerns about lead release during degradation or disposal [89]. Mitigation strategies include lead-sequestration chemistries and the development of lead-free alternatives such as tin- and germanium-based perovskites [89], though these substitutes often suffer from their own instability. In contrast, quantum dots containing cadmium face strict regulatory constraints, prompting a shift toward less toxic compositions such as InP [90] and perovskite nanocrystals [91]. Organic semiconductors frequently rely on chlorinated and aromatic solvents, directing research toward greener solvent systems and redesigned polymer backbones.

Sustainability further encompasses resource availability, energy consumption during synthesis, and end-of-life management. Materials such as III–V semiconductors rely on scarce elements, whereas silicon, carbon-based organics, and earth-abundant chalcogenides offer more sustainable alternatives. Solution-processed perovskites, organics, and quantum dots enable low-temperature fabrication routes with reduced energy demand compared to high-temperature crystal growth. Moreover, emerging recycling procedures, including solvent-based delamination and selective metal extraction, aim to recover valuable components and reduce waste. Finally, comprehensive life cycle assessments (LCAs) increasingly guide material selection by quantifying the carbon footprint, water usage, and environmental impact from raw material sourcing to final disposal.

Table 4 compares stability, toxicity, and sustainability across different classes of materials.

### 3.6. Standardized Stability Measurements

Long-term stability and environmental durability remain among the most critical challenges for the successful commercialization of perovskite-based and hybrid optoelectronic devices. To ensure comparability and reliability across different laboratories and material classes, standardized testing protocols have been developed.

For conventional inorganic semiconductors such as crystalline silicon and III–V compounds, the IEC 61215 [92] series remains the international standard. It includes tests such as thermal cycling (−40 °C to +85 °C), damp heat (85 °C/85% RH for 1000 h), humidity–freeze, UV preconditioning, and mechanical load, which have enabled silicon PV modules to achieve warranties of 25–30 years [93].

For emerging materials such as metal-halide perovskites, organic semiconductors, and hybrid systems, the International Summit on Organic Photovoltaic Stability (ISOS) protocols are widely adopted. These protocols are modular and include ISOS-L (light soaking), ISOS-T (thermal stress), ISOS-D (dark storage), ISOS-H (humidity), and ISOS-V (voltage bias), allowing for the evaluation of degradation mechanisms such as ion migration, phase segregation, and moisture-induced decomposition [94].

Recent advances demonstrate encouraging progress toward meeting industrial standards. For instance, narrowband perovskite photodetectors fabricated via aerosol–liquid–solid spraying exhibited excellent operational stability, maintaining performance after more than 500 on/off illumination cycles in ambient air with around 60% relative humidity without encapsulation [80]. Similarly, lead-free double perovskite Cs_2_AgBiBr_6_ single crystals with (111)-preferred orientation showed superior moisture and light endurance, with negligible degradation after 80 days of ambient exposure, attributed to higher ion migration activation energy and lower defect density compared to other facets [82]. To facilitate the comparison between material classes, Table 5 summarizes the main standardized protocols and their typical stress conditions.

The adoption of these standardized protocols, combined with advanced encapsulation strategies and lead-free compositions, is essential to bridge the gap between laboratory experiments and industrial deployment.

## 4. Solar Energy Harvesting Devices

### 4.1. Photovoltaics

**Figure 1:**
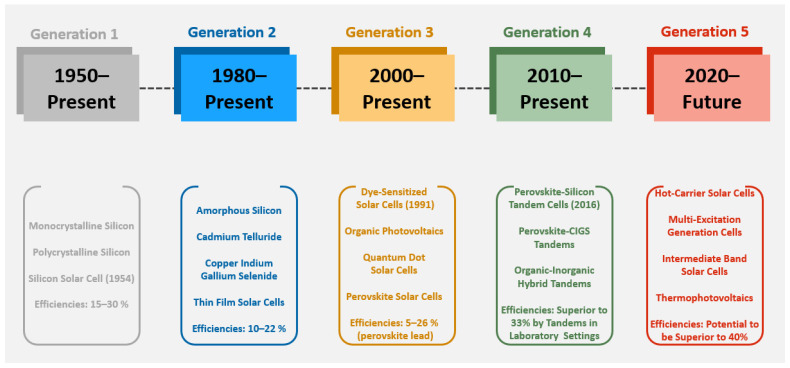
Timeline of the PV technology evolution.

Photovoltaics (PV) technologies remain the most mature and widely deployed approach for converting solar energy into electricity. Their evolution reflects many years of progress in semiconductor physics, materials engineering, and device architecture. This section outlines the main PV categories, including crystalline, thin-film, tandem, and multijunction technologies, highlighting their operating principles, benefits, and limitations. Figure 1 shows the timeline of PV technology evolution.

The evolution of photovoltaic technology is commonly divided into five generations, each representing major shifts in materials, design strategies, and performance goals. The first generation is dominated by crystalline silicon solar cells, including monocrystalline and polycrystalline wafers, which have served as the backbone of the solar industry since the 1950s. These cells offer high efficiency, excellent reliability, and long operational lifetimes; however, their manufacturing requires energy-intensive processes and relatively thick semiconductor wafers, contributing to higher production costs. The second generation introduced thin-film technologies such as amorphous silicon, cadmium telluride (CdTe), and copper indium gallium selenide (CIGS). These devices use extremely thin absorber layers deposited on glass, metal, and flexible substrates, significantly reducing material consumption and enabling lightweight and flexible modules. Although cheaper to produce, thin-film cells often exhibit lower efficiencies and may face stability or toxicity concerns depending on the material system. The third generation encompasses emerging photovoltaic concepts designed to overcome the efficiency and cost limitations of earlier technologies. This group includes dye-sensitized solar cells, organic photovoltaics, quantum dot solar cells, and early-stage metal halide perovskite solar cells.

These technologies emphasize low-temperature, solution-processed fabrication, tunable optical properties, and compatibility with flexible substrates. Nonetheless, many still struggle with long-term stability and large-scale manufacturability. The fourth generation builds on these innovations by combining organic and inorganic materials in hybrid and tandem architectures, with perovskite–silicon tandems becoming the most prominent example. By stacking multiple absorber layers with complementary bandgaps, these devices capture a broader portion of the solar spectrum and surpass the efficiency limits of single-junction cells, achieving the highest laboratory efficiencies while maintaining pathways toward scalable production. Finally, the fifth generation explores advanced and experimental photovoltaic concepts that aim to redefine solar-energy conversion. These include hot-carrier solar cells, multi-exciton generation devices, intermediate-band structures, and thermophotovoltaic systems, all of which seek to exceed the Shockley–Queisser limit through quantum engineering, innovative photonic structures, or advanced thermal management. Although still in early research stages, these next-frontier technologies represent the long-term vision for ultra-high-efficiency solar energy harvesting.

#### 4.1.1. Crystalline Photovoltaics

Crystalline silicon (c-Si) PV accounts for more than 90% of the global market due to its reliability, long operational lifetimes, and well-established manufacturing procedures. Monocrystalline silicon has high purity and a uniform crystal structure, enabling long carrier diffusion lengths and high efficiency [95]. These exceed 22% in commercial modules, with laboratory devices reaching 26.7%. The fundamental benefits of monocrystalline silicon are elevated stability, low toxicity, and compatibility with advanced passivated contact configurations [96]. On the other hand, its limitations involve energy-intensive wafer manufacturing and rigid form factors. Polycrystalline silicon offers lower manufacturing costs due to simpler casting processes. Its grain boundaries introduce recombination sites, reducing efficiency when compared to monocrystalline cells [97]. The typical efficiencies of polycrystalline silicon are around 17% [98]. The main beneficial features of polycrystalline silicon are large-scale enhanced durability and cost-effectiveness.

#### 4.1.2. Thin Film Photovoltaics

Thin-film PV technologies use absorber layers only a few micrometers thick, reducing material consumption and enabling production of flexible and lightweight modules. Amorphous silicon allows low-temperature deposition (up to ~200 °C) on glass, metal, and plastic substrates. One of the most commonly used deposition methods is Plasma-Enhanced Chemical Vapor Deposition (PECVD) [99]. It has a high defect density, which limits single-junction efficiencies to between 6% and 10% [100]. Amorphous silicon thin films are used in portable electronics, solar textiles [101], flexible sensor platforms [102], flat-panel displays [103], and detectors [104].

Furthermore, cadmium telluride has a direct bandgap (~1.45 eV) that enables strong absorption and elevated current generation. CdTe thin-film solar cells reach efficiencies above 20% [105]. Their benefits include lower material usage and compatibility with low-cost deposition techniques, making them very suitable for large-scale deployment [106]. Nonetheless, limitations related to the limited availability and rising cost of tellurium, as well as cadmium toxicity, pose challenges for widespread deployment [107]. Copper indium gallium selenide (CIGS) exhibits a high absorption coefficient, a tunable direct bandgap (1.0–1.7 eV), long-term thermal stability, and good electronic properties in thin-film form [108]. Laboratory solar cells based on this material have already reached an efficiency of around 23.4% [109]. The main limitation is its reliance on indium and gallium, which are scarce elements.

#### 4.1.3. Tandem Photovoltaics

As single-junction technologies approach their theoretical efficiency limits, tandem architecture has emerged as a key strategy to further enhance solar energy conversion. Tandem solar cells stack multiple absorber layers with complementary bandgaps to capture a broader portion of the solar spectrum. Perovskite–silicon tandem devices can reach efficiencies of up to 34.9% using laboratory cells [110]. The perovskite top cell absorbs high-energy photons, while the silicon bottom cell captures lower-energy photons. Key benefits include cost-effective processing and compatibility with existing silicon infrastructure. The main challenges include perovskite stability, conformal deposition on textured silicon surfaces, and multilayer integration complexity [111]. All-perovskite tandems benefit from bandgap tunability, enabling optimal pairing of wide-bandgap (WBG) and narrow-bandgap (NBG) absorbers. All-perovskite solar cells can achieve efficiencies exceeding 29% [112].

The main limitations include: the instability of NBG Sn-based perovskites commonly used in the bottom cell, which are highly prone to Sn^2+^ oxidation that degrades the absorber, increases defect density, and reduces device lifetime; WBG perovskites (top cell), which often suffer from significant open-circuit voltage (VOC) deficits that limit overall efficiency; non-standardized and inefficient Charge Recombination Layers (CRLs), which often introduce resistive losses and interfacial recombination, reducing the tandem performance [113]; and challenges associated with large-area manufacturing [114]. Despite their impressive efficiencies, the scalability and long-term stability of tandem devices remain key barriers to commercialization.

#### 4.1.4. Multijunction Photovoltaics

Multijunction solar cells use three or more semiconductor layers to approach and even exceed the theoretical efficiency limits of single-junction devices. The III–V multijunction cells reach efficiencies above 39% under one-sun illumination and over 47% under concentrated sunlight [115]. The most commonly used materials are the GaInP, GaAs, and InGaAs.

The main benefits include superior thermal stability and high power conversion efficiency (PCE) [116]. These technologies are widely used in space applications and concentrator photovoltaics (CPV). In QD and nanostructure-enhanced multijunction cells, the QD layers enable intermediate-band formation and spectral adjustment, and the nanostructures improve light trapping and carrier extraction [117]. The main limitations of these solar cells are their high manufacturing cost and structural complexity. Table 6 summarizes the benefits, limitations, and applications of these PV technologies.

### 4.2. Perovskite and Hybrid Photovoltaics

Metal-halide perovskites have rapidly transformed emerging photovoltaic technologies, evolving from modest initial efficiencies to laboratory records exceeding 26% in just over a decade [118]. Their main advantages include a tunable bandgap (1.2–2.3 eV) through halide and cation substitution, enabling single-junction and tandem designs, and a high absorption coefficient that allows ultrathin (<1 μm) absorber layers with strong light-harvesting capability. Furthermore, these materials exhibit long charge carrier diffusion lengths and large effective surface areas [119]. Although metal-halide perovskites exhibit relatively high ionic conductivity, which plays a role in defect passivation and self-healing, this same property also drives ion migration processes that act as degradation pathways. In terms of fabrication, metal-halide perovskites can be produced using cost-effective methods such as solvothermal synthesis, Ligand-Assisted Reprecipitation (LARP), CVD, and hot-injection techniques [120]. Regarding perovskite nanocrystals and hybrid systems, the synthesis of CsPbBr_3_/Sn–TiO_2_ nanocrystals serves as a representative example [121,122].

PbBr_2_ is dissolved in a high-boiling solvent in the presence of oleic acid and oleylamine under an inert atmosphere until a clear solution is obtained. A pre-heated Cs-oleate solution is then swiftly injected, leading to rapid nucleation and growth of the CsPbBr_3_ nanocrystals. After a short growth period, the reaction is quenched by cooling and adding a polar anti-solvent, followed by centrifugation and redispersion in a non-polar solvent such as toluene. To form the CsPbBr_3_/Sn–TiO_2_ core–shell nanocrystals, the prepared CsPbBr_3_ dispersion is mixed with titanium and tin precursors (e.g., titanium alkoxide and Sn (IV) compounds). In solvothermal and low-temperature routes, the nanocrystals are often stabilized with additional ligands, such as trioctylphosphine, to improve their resistance to hydrolysis.

The mixture is then transferred into a sealed autoclave and heated at a moderate temperature (typically well below the degradation point of CsPbBr_3_) to promote the controlled hydrolysis and condensation of the Ti/Sn precursors on the nanocrystals, yielding a Sn-doped TiO_2_ shell. After the solvothermal treatment, the product is collected by centrifugation, washed to remove unreacted precursors, and redispersed and dried, giving core–shell CsPbBr_3_/Sn–TiO_2_ nanocrystals for photocatalytic and optoelectronic experiments. CsPbBr_3_ and Cs_4_PbBr_6_ nanocrystals can be obtained within the same colloidal system by tuning the Cs:Pb:Br stoichiometry, ligand environment, and reaction temperature. In a typical one-pot hot-injection synthesis, PbBr_2_ is dissolved in octadecene with oleic acid and oleylamine under an inert atmosphere. The injection of Cs-oleate at elevated temperature produces lead-bromide perovskite nuclei; when the Cs:Pb ratio is close to stoichiometric and the ligand environment is relatively balanced (OA/OAm), the dominant phase is CsPbBr_3_, yielding strongly luminescent green-emitting nanocrystals. Under Cs-rich conditions (higher Cs-oleate loading, a stronger acid environment, or extended aging), the system shifts toward the zero-dimensional Cs_4_PbBr_6_ phase. In practice, CsPbBr_3_/Cs_4_PbBr_6_ composite nanocrystals [123,124] are obtained by choosing intermediate conditions, for instance, slightly Cs-rich compositions and moderate temperatures, so that both phases nucleate and grow concurrently. Finally, post-synthesis treatments such as adjusting the ligand ratio, mild heating, and adding more Cs-oleate can further convert CsPbBr_3_ into Cs_4_PbBr_6_.

Furthermore, PbBr_3_/Cs_4_PbBr_6_ core–shell nanostructures incorporating a metal-halide shell offer enhanced stability and passivation, as synthesized in the work of Ye et al. [125]. CsPbBr_3_/Cs_4_PbBr_6_ heterostructures were coated with PbS shell nanocrystals using a solvothermal approach. The resulting CsPbBr_3_/Cs_4_PbBr_6_@PbS nanocrystals exhibited strong fluorescence in both the visible and near-infrared regions when excited at 365 nm. Importantly, these core–shell nanocrystals showed significantly improved thermal and humidity stability compared with bare CsPbBr_3_ nanocrystals. Surface passivation by the PbS shell enabled the heterostructures to maintain fluorescence at much higher temperatures, as their emission persisted until nearly 200 °C, whereas the fluorescence of CsPbBr_3_ nanocrystals alone was fully quenched at around 100 °C. Additionally, the PbS shell effectively mitigated water-induced degradation, allowing the CsPbBr_3_/Cs_4_PbBr_6_ nanocrystals to retain their fluorescence even after several months of exposure to ambient air. On the other hand, mesoporous perovskites use titanium dioxide and alumina scaffolds to enhance the charge extraction capability by leveraging the electron-injecting capability of titanium dioxide and the structural templating role of alumina. Titanium dioxide facilitates efficient electron injection and charge collection due to its interconnected mesoporous network, whereas alumina provides a non-injecting scaffold that influences perovskite crystallization but reduces photocurrent when used in excess [126].

Moreover, hybrid titanium dioxide and alumina scaffolds allow tuning of the interfacial charge dynamics, while the incorporation of titanium dioxide nanorods further improves the connectivity and extraction efficiency [127]. In terms of device architectures, two main planar configurations dominate perovskite solar cell design: n–i–p (regular) and p–i–n (inverted) structures. Both rely on a thin intrinsic perovskite absorber layer sandwiched between the selective charge transport layers but differ in the sequence of charge extraction. This structural flexibility enables the precise adjustment of the interfacial energetics, reduction in recombination losses, and optimization of charge extraction pathways, all of which are essential for achieving power conversion efficiencies above 25%. The n–i–p configuration, often referred to as the regular structure, has historically led to efficiency records due to its strong electron extraction capability and compatibility with high-quality electron transport materials. In contrast, the p–i–n (inverted) configuration has gained increasing attention because it offers reduced hysteresis, smoother interfaces, and better compatibility with low-temperature processing, making it particularly suitable for flexible substrates and scalable manufacturing.

**Figure 2:**
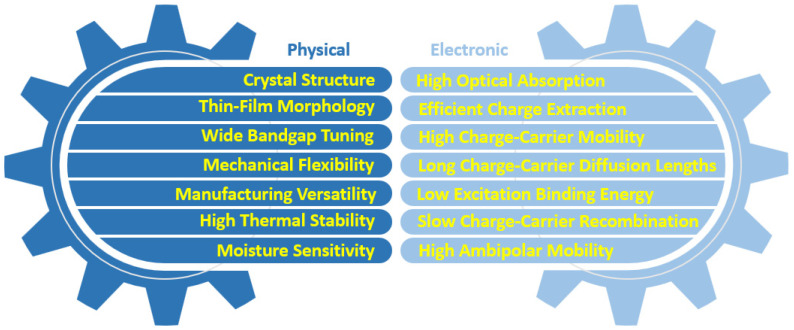
Main physical and electronic properties of the perovskite solar cells.

With the recent advances in interface engineering and transport layer materials, both architectures deliver comparably enhanced efficiencies. Together, these two configurations dominate perovskite solar cell design by balancing efficiency, stability, and manufacturability, offering multiple pathways for optimization in single-junction, tandem, and devices with large areas. Figure 2 presents the fundamental physical and electronic properties of perovskite solar cells.

Thermal stability has been significantly improved through compositional engineering, particularly by incorporating formamidinium and mixed cations (e.g., formamidinium–cesium) in hybrid metal halide perovskites [128], leading to increased power conversion efficiencies and extended operational lifetimes. In addition, hybrid perovskite–silicon tandem cells combine the strengths of both materials to surpass the Shockley–Queisser limit for single-junction devices [110]. In this configuration, the perovskite top cell absorbs high-energy photons (blue–green spectrum), while the silicon bottom cell captures lower-energy photons (red–infrared spectrum). These complementary bandgaps maximize overall photon utilization.

The three following configurations dominate:

- Monolithic (two-terminal) tandems, offering compact integration and high efficiency through current-matched subcells.
- Mechanically stacked (four-terminal) tandems, enabling independent optimization of each subcell without current-matching constraints.
- Interdigitated Back-Contact (IBC) silicon/perovskite tandems, which suppress front-side shading and improve voltage- and current-matching properties.

**Figure 3:**
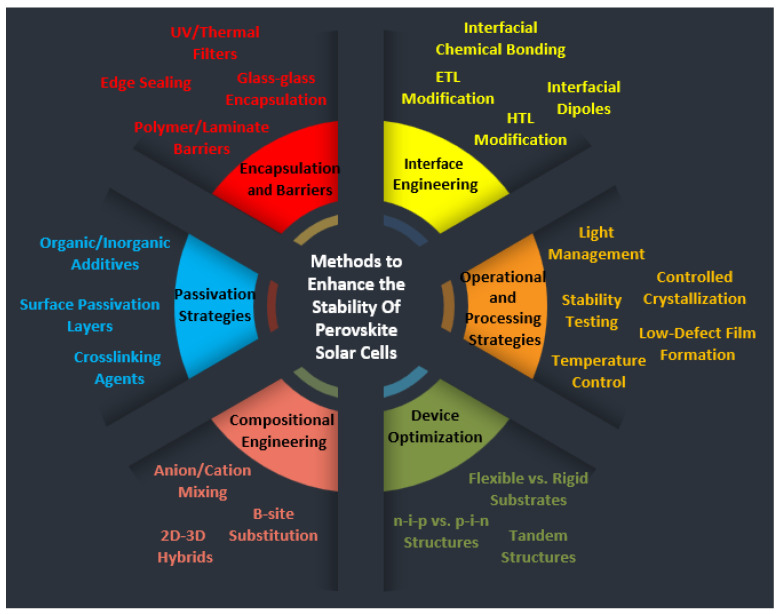
Fundamental areas of actuation and methods to enhance the stability of the perovskite solar cells.

Laboratory tandem efficiencies have approached 35% [110], driven by advances in interfacial recombination layers, optical management, and conformal perovskite deposition on textured silicon. The remaining challenges are long-term perovskite stability, interlayer recombination, and scalable manufacturing. Perovskite–organic hybrid photovoltaics [129] combine the strong absorption and high carrier mobility of perovskites with the flexibility and tunability of organic semiconductors. Figure 3 summarizes the main strategies used to enhance the stability of perovskite solar cells.

Stability remains the central barrier to commercialization. The strategies span materials engineering, interface design, and encapsulation:

- The partial substitution of organic cations with cesium and the incorporation of mixed halides eliminate phase segregation, improve thermal stability, and reduce moisture ingress.
- Additive engineering using polymers, ionic salts, and cross-linkers strengthens the perovskite lattice and reduces the formation of defects.
- Interface passivation with self-assembled monolayers, small molecules, and fullerene derivatives reduces non-radiative recombination and protects against chemical degradation. An illustrative example is the use of C_60_Cl_6_ [130], which slows crystallization, passivates grain boundaries, suppresses Sn^2+^ oxidation, and increases efficiency from 10% to 13.3% in Sn-based perovskites.
- Inorganic transport layers such as NiO_x_ and SnO_2_ replace unstable organic layers, improving thermal stability and reducing ion migration.
- Encapsulation via glass–glass lamination, barrier films, and UV-filtering layers protects against moisture, oxygen, and photo-induced degradation.

**Figure 4:**
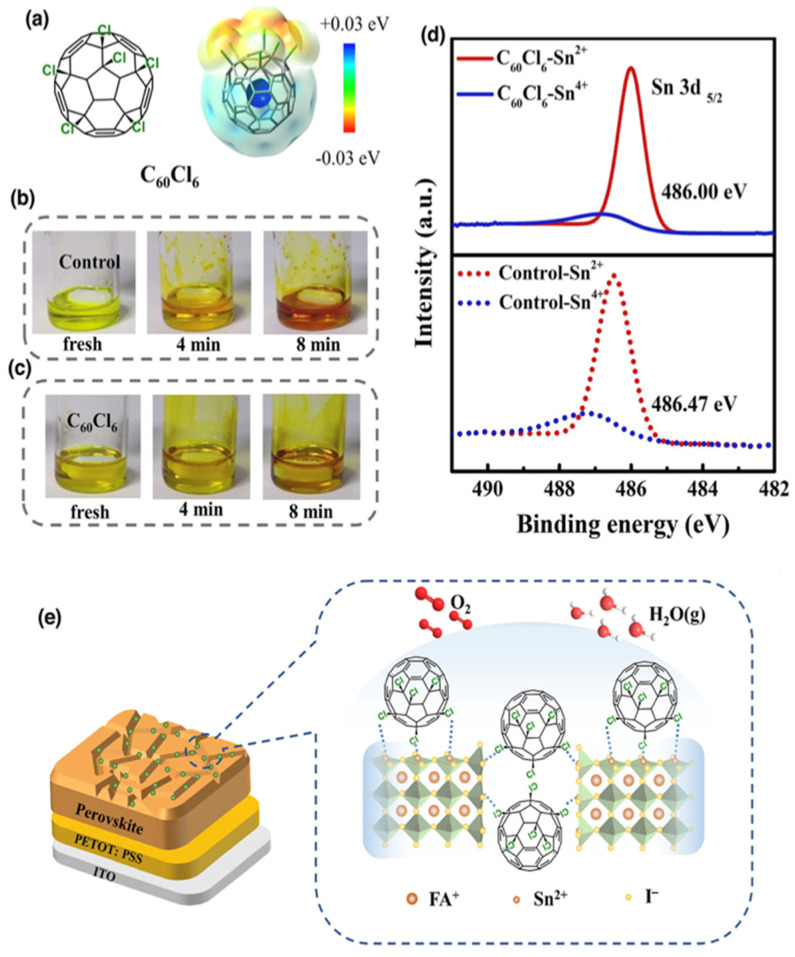
(**a**) Molecular structure and electrostatic potential of the C_60_Cl_6_. Images of perovskite solution: (**b**) without and (**c**) with C_60_Cl_6_ exposed to the air at different times, (**d**) X-ray photoelectron spectroscopy spectra of Sn 3d_5/2_ in control and doping C_60_Cl_6_-based perovskite film, (**e**) C_60_Cl_6_-assisted strategy for device performance enhancement [130].

In work [130], the authors introduced a cholorofullerene C_60_C_l6_, bearing six chlorine atoms on the C_60_ cage, to regulate perovskite crystallization and passivate grain-boundary defects in tin-based perovskite solar cells. Strong chemical interactions between C_60_Cl_6_ and perovskite precursors effectively slow the conversion to perovskite crystals, enabling the formation of high-quality Sn-based perovskite films. Moreover, C_60_Cl_6_ residing at surfaces and grain boundaries not only mitigates defect states but also “stitches” grain boundaries, enhancing the resistance to moisture and oxygen and suppressing the detrimental oxidation of Sn^2+^ to Sn^4+^. As a result, the devices incorporating C_60_Cl_6_ exhibit a substantial efficiency improvement, increasing from 10% to 13.3%, along with markedly enhanced operational stability. Figure 4 shows the molecular structure and electrostatic potential of the cholorofullerene used and other important details of the work.

Replacing unstable organic transport layers with more robust inorganic alternatives (e.g., NiOx, SnO_2_) further enhances thermal and operational stability. Energy-level alignment at interfaces also minimizes ion migration and hysteresis. Finally, encapsulation and device-level protection play a crucial role in long-term durability. Barrier films, glass–glass lamination, and edge-sealing materials prevent moisture and oxygen ingress, while UV-filtering layers mitigate photo-induced degradation. Together, these strategies enable perovskite solar cells to maintain high efficiency while approaching the stability requirements for commercial deployment. Perovskite–organic hybrid PV devices exhibit enhanced thermal stability and broadened spectral absorption due to the synergistic effects of 2D perovskites, polymer interlayers, and organic donor–acceptor materials. The incorporation of 2D perovskite structures significantly improves device robustness, as their layered configurations suppress ion migration, which is a major degradation pathway in conventional 3D perovskites, thereby enhancing long-term operational stability [131]. The polymer interlayers further enhance stability by regulating crystallization, passivating defects, and forming protective networks that inhibit halide and metal ion diffusion while improving interfacial charge transport [132]. These polymers also improve film morphology and reduce recombination losses, which is critical for maintaining performance under thermal stress [133].

In addition, organic donor–acceptor materials extend the spectral coverage by providing tunable bandgaps and efficient light absorption in regions where perovskites are less effective, enabling more comprehensive photon harvesting and improved photovoltaic efficiency [134]. Together, these approaches establish perovskite–organic hybrid PVs as a promising route toward high-performance solar energy technologies. These systems are particularly attractive for semitransparent solar cells for window applications.

For example, Alkhudhari et al. [135] introduced self-organized poly(N-isopropyl acrylamide) microgels as additives in semitransparent perovskite solar cells. These polymers formed a two-dimensional hexagonal nanopore array when deposited from an aqueous solution, which can guide perovskite crystal growth. AFM and SEM characterization confirmed the formation of hexagonal nanopore templates that promoted larger perovskite grains, likely due to interactions between microgel RCOOH groups and the perovskite that slowed crystallization. Using a 15 wt% mixed-cation perovskite (MAFAPbI_3_) with 1.5% polymer microgels, the mesoporous semitransparent device achieved approximately 28.7% Average Visible Transmittance (AVT) and 11.6% power conversion efficiency, compared to 6% for control devices. The resulting Light Utilization Efficiency (LUE) was approximately 2.6%. This study represents the first demonstration of microgels incorporated into the perovskite precursor solution via a single-step process, improving the AVT while supporting scalable manufacturing. Mixed 2D/3D perovskite systems also offer significant advantages for semitransparent solar cells. Incorporating 2D perovskite components into a 3D matrix helps control crystallization, producing smoother and more uniform thin films that are suitable for light transmission.

The 2D layers also widen the bandgap and reduce visible light absorption, increasing AVT without severely compromising efficiency. At the same time, 2D phases act as protective barriers that enhance moisture and thermal stability, which is an important benefit for the thinner, more exposed films used in semitransparent devices. As a result, mixed 2D/3D formulations provide an effective balance between transparency, performance, and stability in semitransparent perovskite solar cells.

For instance, Zou et al. [136] incorporated mixed 2D/3D perovskites into semitransparent devices by adding 2% of a 2D perovskite (3TMA)_2_PbCl_4_ to a Cs_0_._13_FA_0_._87_Pb(I_0_._87_Br_0_._13_)_3_ solution. This small 2D fraction significantly improved film quality, producing morphology with grain sizes of ~500 nm compared to ~100 nm for pure 3D films. The enhancement was attributed to the interactions between the amino groups of the 2D perovskite and the 3D lattice, which slowed nucleation and improved crystal growth. The devices were fabricated in an inverted architecture (glass/ITO/MeO-2PACZ/perovskite/C_60_/SnO_2_/IZO/Silver grid) with a 190 nm absorber layer optimized for transparency and efficiency. The best-performing device achieved 30.1% AVT and 14.1% PCE, corresponding to a high LUE of 3.1%, and retained 95% of its efficiency after 500 h under ambient conditions.

Hybrid perovskite–quantum dot (QD) photovoltaics extend absorption into the infrared using materials such as PbS QDs [137], enabling spectral tuning and improved charge extraction. These systems also show potential for intermediate-band solar cells, though challenges remain relative to environmental stability and ion migration. Table 7 summarizes the benefits, efficiency potential, and challenges of the major perovskite photovoltaic technologies, including single-junction perovskites, perovskite–silicon tandems, perovskite–organic hybrids, and perovskite–QD systems. Collectively, these approaches demonstrate the versatility of perovskite materials and their potential to deliver high-efficiency, low-cost, and scalable solar energy solutions.

A deeper mechanistic understanding of perovskite and hybrid photovoltaic operation reveals that their exceptional performance arises from the interplay between electronic structure, ionic dynamics, and interfacial chemistry. Metal-halide perovskites possess a soft polar lattice in which dynamic disorder and intense spin–orbit coupling shape the band structure and suppress deep trap formation, enabling long carrier lifetimes even in polycrystalline films [138]. This defect tolerance is further enhanced by the antibonding character of the valence band, which energetically disfavors the formation of deep recombination centers. However, the same lattice softness facilitates ion migration, particularly of halides and A-site cations, which leads to field screening, hysteresis, and long-term degradation under bias and illumination [139]. Understanding the coupling between ionic motion and electronic transport remains a fundamental challenge for stabilizing perovskite devices.

Hybrid nanocrystal systems introduce additional mechanistic considerations. In CsPbBr_3_/Sn–TiO_2_ and CsPbBr_3_/Cs_4_PbBr_6_@PbS heterostructures, charge extraction is governed by interfacial band alignment, ligand chemistry, and quantum-confinement effects. The inorganic shells (e.g., PbS, TiO_2_) passivate the surface traps and modulate the carrier transfer pathways by altering the interfacial dipoles and suppressing halide-vacancy formation [140]. These mechanisms explain the enhanced photostability and thermal robustness observed in core–shell nanocrystals compared with bare perovskite nanocrystals.

Comparing device architectures highlights distinct mechanistic tradeoffs. n–i–p structures typically achieve higher open-circuit voltages due to efficient electron extraction and well-established electron-transport layers, yet they are more susceptible to UV-induced degradation of titanium dioxide and interfacial photocatalysis. p–i–n architectures, by contrast, benefit from reduced ion accumulation at contacts, smoother interfaces, and low-temperature processing, making them more compatible with flexible substrates and scalable manufacturing [141]. Mechanistically, p–i–n devices often exhibit reduced interfacial recombination because their hole-transport layers form more favorable energetic alignments with the perovskite valence band.

Despite rapid progress, several critical limitations continue to impede commercialization. First, environmental instability remains a major barrier: perovskites degrade through moisture-induced hydrolysis, oxygen-driven superoxide formation, thermal phase transitions, and UV-accelerated lattice decomposition [142]. Even with mixed-cation and mixed-halide engineering, phase segregation and halide redistribution can occur under prolonged illumination. Second, interfacial recombination persists as a dominant loss mechanism. Imperfect band alignment, trap-rich grain boundaries, and chemically reactive transport layers accelerate non-radiative recombination and reduce device lifetimes [143]. Third, scalability challenges arise from the difficulty of maintaining uniform crystallization, defect passivation, and film morphology over large areas. Many deposition methods at laboratory scale cannot reproduce high-quality films in roll-to-roll and vapor-assisted processes [144].

Hybrid perovskite–organic and perovskite–quantum dot systems introduce further complexities. Organic interlayers can enhance flexibility and spectral tunability but may introduce thermal or chemical instability at interfaces. Furthermore, quantum dot additives improve infrared absorption and passivation but suffer from ligand desorption, oxidation, and interfacial diffusion under operational conditions [145]. These issues underscore the need for deeper mechanistic insight into interfacial chemistry, ion–molecule interactions, and multiscale degradation pathways.

Looking forward, the path to commercialization will require coordinated advances in materials design, interface engineering, scalable deposition, and encapsulation strategies. Emerging approaches such as 2D/3D heterostructures, cross-linked perovskite lattices, and self-assembled passivation layers show promise for suppressing ion migration and enhancing environmental stability [146]. Continued progress in these areas will determine whether perovskite and hybrid photovoltaics can transition from laboratory demonstrations to reliable and bankable solar technologies.

### 4.3. Concentrator Photovoltaics and Light Management

**Figure 5:**
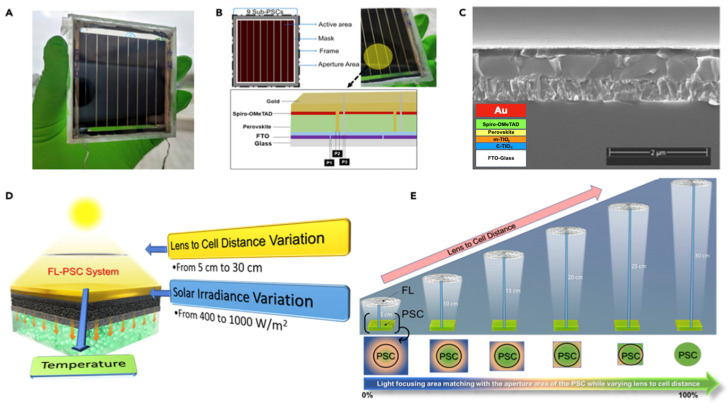
Development of a large-area perovskite solar cell module and interpretation of the photovoltaic performance in concentrated sunlight PSC: (**A**) Photograph of the encapsulated large-area PSC module, (**B**) Schematic representation of the module structure with nine sub-cells connected in series, (**C**) Cross-sectional SEM image of a sub-cell in the PSC module, (**D**) Operational testing view of the FL-PSC system, and (**E**) Schematic representation of the PV performance evaluation of FL-PSC system under various lens-to-cell distances [147].

Concentrator photovoltaics (CPV) and advanced light management are effective options for pushing solar cell performance beyond the limits of traditional flat-plate designs. By controlling how light is captured, guided, and concentrated, these approaches enable higher efficiencies, reduced material usage, and improved performance under diverse operating conditions. They are particularly valuable for high-efficiency III–V devices, thin-film technologies, large-area perovskite solar modules [147], as illustrated in Figure 5, and hybrid systems.

**Figure 6:**
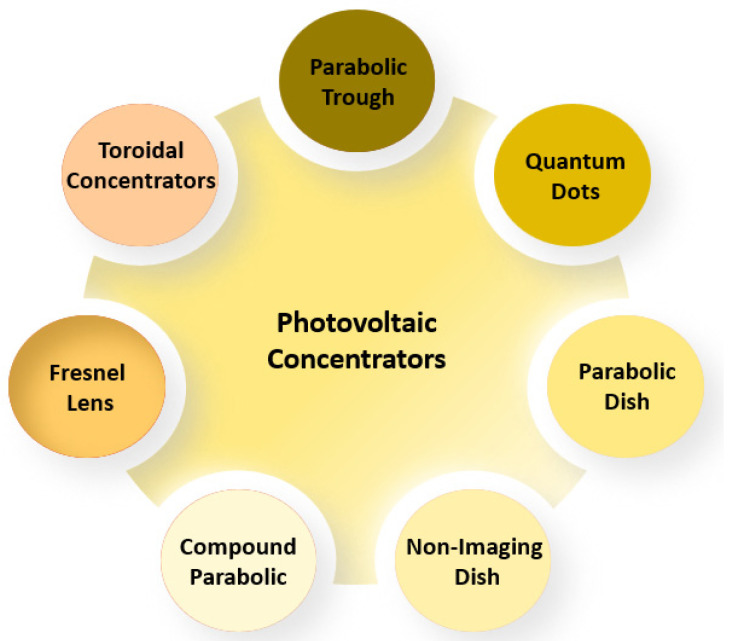
Main PV concentrators.

CPV systems use optical components such as lenses and mirrors to concentrate sunlight onto high-efficiency solar cells. By increasing photon flux, CPV systems enable smaller, high-performance cells to generate significantly more power [148]. Optical concentrators, including Fresnel lenses and parabolic or parabolic-trough reflectors, focus sunlight onto a small-area solar cell, with typical concentration ratios ranging from around 10 times (low concentration) to over 1000 times (high concentration) [149]. Figure 6 shows the main PV concentrators.

Multijunction III–V solar cells are commonly used in CPV systems due to their superior performance under high irradiance. These systems achieve efficiencies superior to 47% under concentrated sunlight [150]. For example, Geisz et al. [150] reported a record efficiency of 47.1% using a monolithic, six-junction Inverted Metamorphic (IMM) solar cell operating under 143 suns. When optimized for the global spectrum, a related design achieved approximately 39% efficiency under one-sun illumination. These six junctions were engineered with near-optimal bandgaps using III–V semiconductor alloys. Their development required suppressing threading dislocations in lattice-mismatched III–V materials, avoiding phase segregation in metastable quaternary alloys, and understanding dopant diffusion in complex multilayer structures. Further reductions in series resistance could push efficiencies beyond 50%. An additional advantage is the reduced semiconductor usage, as only a small active area is required, lowering the cost of expensive III–V materials.

CPV systems also exhibit strong performance in regions with high direct normal irradiance (DNI) [151]. However, the following limitations remain:

- Precise solar tracking requirements.
- Significant thermal management needs due to heat generation [152].
- Poor performance under diffuse and cloudy weather conditions.
- Optical losses caused by dust accumulation and material absorption/reflection [153].

Applications include hybrid wind–solar power plants [154], utility-scale installations [155], and space photovoltaics [156], where reduced mass and high efficiency are critical. Advanced light management strategies, such as anti-dust coatings and antireflection coatings (ARCs) [157], enhance absorption, reduce reflection losses at the air–semiconductor interface, and improve photon utilization. These approaches are essential for thin-film devices, perovskites, organics, and nanostructured PVs in which absorber layers are ultrathin. Biomimetic nanostructured “moth-eye” surfaces provide broadband and omnidirectional antireflection [158]. Micro- and nanotexturing scatter incident light, increasing the optical path length within the absorber. For example, silicon wafers commonly use a pyramid texture to reduce reflection and enhance absorption [159]. Thin-film devices use textured substrates and scattering layers to compensate for their limited thickness. Metallic nanoparticles can induce localized surface plasmon resonances, enhancing near-field absorption. Photonic crystals and metasurfaces [160] enable wavelength-selective trapping by controlling light propagation.

These structures are especially useful for ultrathin perovskite and organic solar cells. Back reflectors, such as aluminum layers and dielectric mirrors [161], redirect unabsorbed light back into the active layer. Waveguiding structures confine light within the absorber, improving absorption at oblique angles [162]. Furthermore, Distributed Bragg Reflectors (DBRs) [163] enhance spectral selectivity, particularly in tandem devices. Dichroic mirrors and prisms can split sunlight into spectral bands directed to different subcells, enabling high-efficiency multijunction architectures without strict lattice-matched constraints. They are very useful for hybrid perovskite–silicon and perovskite–organic tandems. Table 8 summarizes the main benefits and limitations of light management strategies for concentrator photovoltaic systems.

### 4.4. Dynamic Thermal Regulation Under Variable Solar Radiation Intensity

In practical smart grid and integrated renewable energy systems, optoelectronic devices operate under highly dynamic environmental conditions, with solar radiation intensity varying significantly due to weather, time of day, seasonal changes, and partial shading. These fluctuations cause rapid temperature swings in the devices, which can severely impact efficiency, stability, and lifetime through increased non-radiative recombination, accelerated ion migration (especially in perovskites), and thermal stress at interfaces. Dynamic thermal regulation refers to procedures that actively or passively adapt the thermal behavior of the device or system in response to real-time operating conditions. The main routes are the following:

- Passive thermal management requires the use of nanostructured surfaces, photonic metamaterials, and radiative cooling layers that dynamically modulate the emissivity and the reflectivity according to temperature and incident solar flux. For example, temperature-responsive materials can increase infrared emission at elevated temperatures to dissipate heat more effectively.
- Active thermal management involves the integration of micro-fluidic cooling channels, phase-change materials (PCMs), and thermoelectric elements controlled by embedded sensors. These systems adjust cooling rates based on the instantaneous irradiance levels.
- Smart AI-driven control based on machine learning models processes data from irradiance sensors, thermal cameras, and device performance metrics to predict temperature profiles and optimize operating parameters (e.g., maximum power point tracking with thermal derating, adaptive biasing, or dynamic shading). This enables real-time decision-making to minimize thermal degradation while maintaining a high energy yield.

Recent demonstrations highlight the potential of these strategies. Perovskite-based multispectral photodetectors [82,164,165] and intelligent cameras [80] have shown robust performance under varying illumination conditions, partly due to optimized thermal stability through material engineering and computational compensation. Similarly, oriented lead-free double perovskites exhibit enhanced thermal and moisture stability, providing a more robust platform for dynamic operating environments [82,164]. Additionally, customized perovskite photodetectors, including multidimensional and dynamic-tracking devices, show promise for operation under variable conditions [165].

The combination of advanced materials, nanostructured thermal interfaces, and AI-predictive control is expected to play a pivotal role in next-generation smart integrated optoelectronic systems, ensuring reliable operation across different sunlight intensities and environmental conditions.

### 4.5. Performance Metrics for Solar Energy Harvesting Devices

Understanding performance metrics is essential for evaluating, comparing, and optimizing solar energy harvesting devices. These parameters describe how efficiently a photovoltaic system converts sunlight into electricity, how it performs under real-world conditions, and how its performance evolves over time. Photovoltaic performance can be evaluated by the following metrics:

- Power conversion efficiency (PCE)—the fraction of incident solar power converted into electrical power. It is determined by the open-circuit voltage (VOC), short-circuit current density (JSC), and fill factor (FF). A high PCE requires intense light absorption, efficient charge separation, and minimal recombination losses.
- VOC is the maximum voltage when no current flows. It is sensitive to recombination losses and energy-level alignment within the device. Higher VOC values indicate reduced non-radiative recombination.
- JSC can be defined as the current generated under illumination when the device is short-circuited. It depends on the absorption spectrum, carrier diffusion length, and light management strategies.
- FF measures the squareness of the current–voltage curve. It is affected by series resistance, shunt pathways, and interface defects.
- External Quantum Efficiency (EQE)—the fraction of incident photons converted into collected charge carriers as a function of wavelength. It provides insight into spectral absorption and carrier collection efficiency.
- T80/T90—the time required for the device efficiency to decrease to 80% or 90% of its initial value. Standard stability tests include thermal cycling, damp heat exposure, and UV irradiation. Operational stability under maximum power point tracking (MPPT) conditions is increasingly emphasized.

### 4.6. Degradation Mechanisms

Photovoltaic (PV) devices degrade through a combination of environmental stressors, intrinsic material instabilities, and interfacial reactions. While the specific pathways differ across the diverse technologies, most degradation phenomena originates from defect formation, interlayer chemical reactions, and the progressive breakdown of contacts and interfaces.

#### 4.6.1. Intrinsic Material and Environmental Degradation

**Figure 7:**
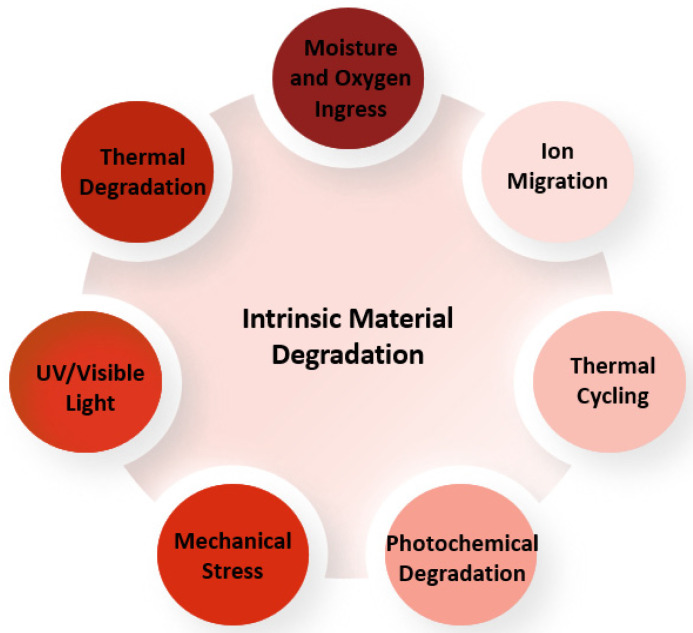
Main intrinsic material and environmental degradation stressors.

Figure 7 summarizes the principal intrinsic and environmental stressors affecting PV materials.

High temperatures promote defect generation, ion migration, and chemical decomposition across perovskites, organic semiconductors, and thin-film chalcogenides. Photochemical degradation driven by high-energy photons breaks chemical bonds, generates reactive species, and alters the electronic structure of absorber and transport layers. UV exposure further degrades polymers, encapsulants, and certain perovskite formulations [166].

Mobile ionic species, such as halides in perovskites, can drift under internal electric fields, producing hysteresis, interfacial degradation, and light-induced phase segregation in mixed-halide compositions [167,168]. Also, moisture and oxygen ingress trigger oxidation, hydrolysis, and structural decomposition, particularly in perovskites and organic materials, which lack intrinsic chemical robustness [169]. Thermal expansion and contraction during diurnal cycling may induce microcracks, delamination, and mechanical fatigue, further accelerating environmental degradation.

#### 4.6.2. Interface and Contact Degradation

Device interfaces and contacts are among the most vulnerable regions in PV architectures. Metal electrodes such as silver and aluminum can diffuse into adjacent layers, forming deep recombination centers and disrupting charge-transport pathways. Barrier layers and alternative contact materials mitigate, but do not eliminate, this risk.

Chemical incompatibilities between adjacent layers frequently lead to defect formation at perovskite/transport-layer interfaces and in organic heterojunctions. The interfacial reactions may produce intermetallic compounds, halide-rich regions, and oxidized species that degrade carrier extraction. Also, the procedures to suppress defect formation involve large-grain growth, grain-boundary and substrate modification, photo-curing, and surface passivation [170,171].

Transport layers—both organic and inorganic—also degrade under thermal stress, moisture exposure, and prolonged illumination. Poor energy-level alignment exacerbates recombination losses, reduces open-circuit voltage (VOC), and accelerates interfacial chemical reactions. These effects are particularly pronounced in devices with mixed-dimensional and chemically reactive interfaces.

#### 4.6.3. Comparative Degradation Across PV Technologies

Table 9 provides a comparative overview of degradation mechanisms across major PV technologies.

- Perovskite devices are dominated by ion migration, phase segregation, and interfacial reactions with metal contacts and transport layers.
- Organic PVs primarily suffer from photo-oxidation, thermal bond scission, and morphological instability.
- Thin-film chalcogenides exhibit defect generation under thermal and light stress, along with contact diffusion.
- Silicon technologies show comparatively slower degradation, driven mainly by light-induced defect activation and contact corrosion.

Together, these degradation pathways determine the practical viability of PV technologies. High efficiency is meaningful only when paired with long-term operational stability, and understanding these mechanisms is essential for designing more durable materials, interfaces, and encapsulation procedures.

## 5. Light-Driven Energy Conversion and Storage

### 5.1. Photoelectrochemical Water Splitting

Photoelectrochemical (PEC) water splitting is one of the most promising solar-to-fuel technologies, enabling the direct conversion of sunlight into hydrogen [172]. By integrating light absorption, charge separation, and catalytic reactions into a single device, PEC systems offer great potential for carbon-neutral storage and a sustainable hydrogen economy. PEC water splitting relies on semiconductor photoelectrodes that absorb sunlight and generate electron–hole pairs. These carriers drive the two half-reactions of the hydrogen evolution reaction (HER) at the photocathode and the oxygen evolution reaction (OER) at the photoanode. A functional PEC device must achieve strong solar absorption, efficient charge separation and transport, fast interfacial charge transfer to catalysts, and chemical stability in aqueous electrolytes [173]. The semiconductor band edges must straddle the redox potentials of water (0 V and +1.23 V vs. RHE) to ensure thermodynamic feasibility.

#### 5.1.1. Photoanode Materials for OER

Photoanodes must withstand oxidative conditions while providing high photovoltage and catalytic activity. Regarding metal oxides, titanium dioxide is highly stable but has a wide bandgap (3.0–3.2 eV) that limits visible light absorption. Hematite is abundant and stable, with a moderate bandgap (~2.1 eV), but suffers from a short hole diffusion length. BiVO_4_ exhibits strong visible light absorption and good band alignment but requires surface catalysts for efficient OER. In terms of III–V semiconductors, GaN, GaAs, and InP have improved optoelectronic properties and efficiencies but require protective coatings to prevent corrosion.

#### 5.1.2. Photocathode Materials for HER

Photocathodes must reduce protons to hydrogen while resisting corrosion. Fundamental examples are p-type semiconductors, which exhibit strong performance with adequate surface passivation and catalysts; Cu_2_O, which has good band alignment but is prone to photo-corrosio; and GaInP_2_ with elevated efficiency in tandem PEC. Catalyst materials include platinum, which is the benchmark HER catalyst, but reliable alternatives include MoS_2_, Ni–Mo alloys, and cobalt.

#### 5.1.3. Device Configurations

**Figure 8:**
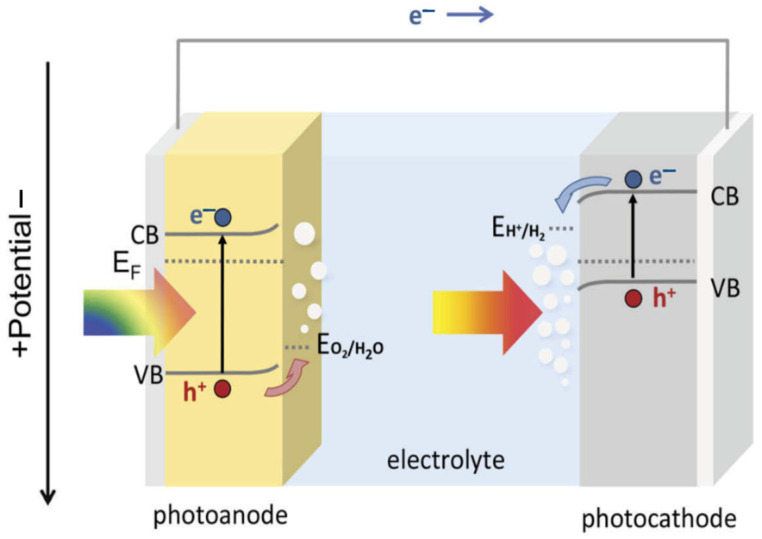
Schematic representation of a PEC/PV tandem cell configuration [175].

Single-absorber PEC cells use one semiconductor to drive both HER and OER. They are simpler but often limited by insufficient photovoltage. Dual-absorber (Z-scheme) systems mimic natural photosynthesis by combining two absorbers with complementary bandgaps [174]. These systems enable higher Solar-to-Hydrogen (STH) efficiencies. Tandem PEC–PV hybrids can pair a PV cell with a PEC electrode [175] as shown in Figure 8.

PV provides additional voltage, enabling high STH efficiencies, with reported values exceeding 20% in prototypes. Moreover, tandem PEC-PV hybrid architectures with the assistance of spectral beam splitters were already reported in the literature in the work performed by Wang et al. [176]. They developed a novel self-biased hybrid PEC system by integrating titanium dioxide and BiVO_4_ photoelectrodes with spectral beam splitters and a PV cell to maximize solar energy utilization and enhance water-splitting performance. Incorporating spectral beam splitters significantly increases the current densities of both self-biased PV–PEC configurations and standalone PEC devices. The self-biased system equipped with beam splitters delivers higher current density than conventional TiO_2_/BiVO_4_-PV configurations, and the intersection of the photoanode and PV current-voltage (I–V) curves shifts closer to the maximum power point of the PV cell. By enabling both the photoelectrode and PV cell to more effectively harvest different portions of the solar spectrum, the beam-splitter-coupled hybrid system achieves an 18.8-fold increase in power output in comparison to a conventional tandem self-biased system. Predictive analysis indicates a hydrogen production rate of 12.1 µmol. h^−1^.cm^−2^, with an STH efficiency improved by factors of 12.4 and 19.9 compared to TiO_2_/BiVO_4_-PV and TiO_2_/BiVO_4_ tandem systems, respectively.

#### 5.1.4. Performance Metrics

The key performance metrics for PEC systems are:

- STH efficiency—overall conversion efficiency from incident solar energy to chemical fuel (hydrogen).
- Faradaic efficiency—fraction of charge carriers that effectively contribute to hydrogen and oxygen evolution reactions.
- Onset potential—voltage at which photocurrent begins, indicating catalytic activity and interfacial quality.
- Stability (T50/T80)—time required for the device to reach 50% and 80% of its initial performance under continuous operation.

#### 5.1.5. Degradation Issues

One of the main degradation mechanisms is photocorrosion, as many semiconductors, such as GaAs and Cu_2_O, degrade under illumination in aqueous environments. To mitigate this effect, protective coatings such as titanium dioxide, alumina, and graphene can be employed. The catalyst particles may also detach, oxidize, or restructure; therefore, strong adhesion and stable supports are critical. Another relevant degradation pathway is electrolyte-induced degradation, as pH and ionic species strongly influence material stability. Alkaline electrolytes improve OER kinetics but may corrode certain materials. Interfacial instabilities also contribute to degradation, as poor band alignment and defective interfaces increase recombination losses and reduce the photovoltage [177].

#### 5.1.6. Advances and Research Directions

Protective coatings deposited via Atomic Layer Deposition (ALD), such as TiO_2_, Al_2_O_3_ and Ta_2_O_5_, have significantly improved the stability of III–V semiconductors in PEC systems [178]. Nanostructured electrodes enhance light absorption and increase the catalytic surface area. Earth-abundant catalysts are reducing reliance on noble metals. Integrated PEC panels are emerging as promising solutions for scalable outdoor operation [179].

#### 5.1.7. Mechanistic Discussion and Limitations

A more detailed mechanistic picture of PEC water splitting highlights the interplay between semiconductor physics, interfacial electrochemistry, and catalytic kinetics. When illuminated, the semiconductor photoelectrodes generate electron–hole pairs whose separation is driven by built-in electric fields, band bending at the semiconductor–electrolyte interface, and catalytic overpotentials. The photogenerated holes at the photoanode must oxidize water through the multistep OER, which involves four Proton-Coupled Electron-Transfer (PCET) steps and the formation of O–O bonds, which are processes that are sluggish and require efficient OER catalysts to suppress surface recombination. Conversely, the electrons at the photocathode drive the HER, which proceeds via Volmer–Heyrovsky or Volmer–Tafel pathways depending on the catalyst and pH. The semiconductor band edges must not only straddle the water redox potentials but also provide sufficient overpotential to overcome the kinetic barriers, making band-edge engineering and surface catalyst integration central to PEC design.

A comparative analysis of material classes reveals distinct tradeoffs. Metal oxides such as TiO_2_, Fe_2_O_3_, and BiVO_4_ offer enhanced chemical stability but suffer from poor charge mobility, short minority-carrier diffusion lengths, and sub-optimal bandgaps that limit Solar-to-Hydrogen (STH) efficiency. The III–V semiconductors, such as GaInP_2_ and GaAs, achieve record STH efficiencies due to their ideal bandgaps and high carrier mobilities, yet their high cost, scarcity, and corrosion susceptibility restrict large-scale deployment. Emerging materials—including 2D transition-metal dichalcogenides, and quantum dot heterostructures—provide tunable optoelectronic properties and strong absorption, but their long-term stability in aqueous electrolytes remains a major barrier. Protective layers such as TiO_2_, Al_2_O_3_, and NiO_x_ deposited by ALD or sputtering can mitigate corrosion but often introduce resistive losses or impede charge transfer, requiring careful optimization of thickness and interfacial energetics.

Hybrid metal-halide perovskites possess strong solar absorption and favorable optoelectronic properties (see Section 4.2), making them attractive candidates for PEC and PEC–PV [180] hybrid systems. However, their use as photoelectrodes is severely limited by intrinsic instability in aqueous electrolytes, where hydration, ion migration, and dissolution rapidly degrade the material. Consequently, perovskite-based PEC devices need robust encapsulation and protective coatings, such as ALD-deposited TiO_2_, Al_2_O_3_, and Ta_2_O_5_, to prevent water ingress and photocorrosion. While perovskite–PEC hybrid architectures show great potential for high-efficiency solar fuel generation, long-term durability remains the primary barrier to practical deployment.

Current PEC systems face several critical limitations. First, interfacial recombination remains a dominant loss pathway: surface trap states, catalyst–semiconductor mismatches, and poor band alignment reduce charge-transfer efficiency and increase the required overpotential. Second, stability under operational conditions—including photocorrosion, catalyst dissolution, and mechanical delamination—continues to limit device lifetimes, especially for high-performance III–V systems. Third, scalability challenges arise from the need for large-area, defect-free photoelectrodes with uniform catalyst coverage and resistant protective coatings. Fourth, mass-transport limitations like bubble formation, electrolyte depletion, and pH gradients can reduce photocurrent at high operating densities, requiring optimized cell geometries and flow-cell architectures. Finally, system-level integration with membranes, gas-separation units, and balance-of-plant components remains underdeveloped, hindering the translation of laboratory devices into practical solar-hydrogen generators.

Recent reviews [181,182,183,184] emphasize that overcoming these limitations will require coordinated advances in semiconductor design, catalyst engineering, protective coating strategies, and device architecture. Integrated PEC–PV hybrid systems, tandem absorbers, and buried-junction architectures are emerging as promising pathways to combine high efficiency with improved stability. However, achieving commercially relevant STH efficiencies greater than 10% with a prolonged lifetime remains an open challenge, underscoring the need for a deeper mechanistic understanding and materials innovation.

### 5.2. Solar Fuels and Artificial Photosynthesis

Solar fuel technologies aim to convert sunlight, water, and carbon-based molecules into chemical fuels such as hydrogen, methanol, ammonia, and hydrocarbons. Artificial photosynthesis translates this concept by mimicking the natural photosynthetic process, which involves absorbing sunlight, separating charge, and driving catalytic reactions, to produce fuels with zero carbon emissions [185]. Together, these approaches represent a crucial bridge between intermittent solar energy and continuous energy demands. Artificial photosynthesis integrates light absorption with semiconductor materials that capture sunlight and generate electron–hole pairs, followed by charge separation and transport. Photogenerated carriers migrate to catalytic sites without recombining. Electrons and holes drive redox reactions such as water reduction to hydrogen, water oxidation to oxygen, carbon dioxide reduction to carbon monoxide, CH_4_, and CH_3_OH, and nitrogen reduction to ammonia.

Carbon dioxide reduction [186] is one of the most challenging and impactful solar fuel pathways, offering a route to carbon-neutral and carbon-negative fuels. The main catalysts and materials employed in carbon dioxide reduction include nanoparticles of copper, silver, and gold for selective carbon monoxide and hydrocarbon formation; molecular catalysts such as porphyrins and bipyridyl complexes with tunable selectivity; semiconductor photocathodes, including gallium phosphide, silicon [187], and perovskites for integrated PEC carbon dioxide reduction [188], and Metal–Organic Frameworks (MOFs) and Covalent Organic Frameworks (COFs) for high surface area and adjustable active sites [189].

### 5.3. Solar-Driven Hydrogen Production Beyond PEC Water Splitting

Beyond the traditional PEC systems, several hybrid approaches exist. One of them is photocatalytic water splitting using semiconductor powders of TiO_2_, g-C_3_N_4_, and SrTiO_3_ dispersed in water [190]. Although this is a simple and scalable process, it usually has lower efficiency than PEC systems. Furthermore, Z-scheme photocatalysis mimics natural photosynthesis by coupling two photocatalysts [191]. This process enables visible light absorption and higher overall efficiency. Integrated photocatalyst sheets provide large-area films combining light absorbers and catalysts. They are promising for scalable hydrogen production without external wiring.

### 5.4. Solar-Driven Nitrogen Fixation

Artificial nitrogen fixation aims to produce ammonia using sunlight instead of the energy-intensive Haber–Bosch process [192]. One promising approach is the Photocatalytic Nitrogen Reduction (PNRR) using metal oxides, metal sulfides, and plasmonic catalysts focused on activating nitrogen under solar irradiation by improving nitrogen adsorption, charge separation, and catalytic active-site engineering. Metal oxides are widely used due to their chemical stability, tunable band structures, and compatibility with heterojunction development. A recently published review [193] highlights that metal oxides serve as supports in Metal-Support Interaction (MSI) systems, where the geometric and electronic effects improve nitrogen activation and suppress competing hydrogen evolution. These oxides help modulate charge transfer and stabilize active metal sites, enhancing the overall PNRR efficiency.

Furthermore, metal sulfides such as CdS, ZnCdS, and CuFeS_2_ offer narrow bandgaps and strong visible light absorption, making them very promising for nitrogen reduction. Their electronic structures facilitate stronger interactions with nitrogen, and MSI-based designs using sulfide supports can significantly enhance catalytic performance [194]. Although much research on sulfides focuses on hydrogen evolution, insights from these studies—such as 3D electronic dimensionality, defect engineering, and photocorrosion control—translate directly to improving nitrogen reduction by enhancing charge mobility and stabilizing active sites [195]. Moreover, plasmonic sulfides, such as CuFeS_2_, have also demonstrated improved photocatalytic activity in reduction reactions, showing how hot-carrier generation and photothermal effects can appreciably accelerate the surface reactions [196]. Other promising routes are PEC nitrogen reduction using tailored photocathodes and bio-inspired catalysts mimicking nitrogenase enzymes [197].

### 5.5. Photocatalytic Carbon Dioxide Reduction

Photocatalytic carbon dioxide reduction is a cornerstone of artificial photosynthesis research, aiming to convert carbon dioxide into valued fuels and chemicals using sunlight as the sole energy input. This process offers a dual environmental benefit: mitigating carbon dioxides emissions while producing carbon-neutral fuels such as carbon monoxide, methane, methanol, formic acid, and other hydrocarbons. Photocatalytic carbon dioxide reduction involves light absorption and exciton generation. Specifically, semiconductor photocatalysts absorb photons and generate electron–hole pairs, followed by charge separation and migration. Electrons move to reduction sites, while holes migrate to oxidation sites to drive water oxidation and surface catalytic reactions. Electrons reduce carbon dioxide through multi-electron, multi-proton pathways, while holes oxidize water to supply protons. The overall reaction must overcome the high thermodynamic stability of carbon dioxide, making catalyst design and charge management of the utmost importance.

The main strategies to improve photocatalytic performance are doping and alloying to adjust band positions for visible light absorption and optimal redox potentials [198], co-catalysts like copper, nickel, and cobalt to reduce activation barriers and improve selectivity [199], passivation layers to suppress the recombination and stabilize reactive surfaces [200], QDs to enhance charge separation and enable size-dependent tuning [201], nanorods and nanosheets to increase surface area and reduce diffusion paths [202], porous supports like MOFs and COFs to increase local carbon dioxide concentration [203], surface functional groups such as amines and pyridines to promote carbon dioxide binding and activation [204], and the use of a dual photocatalyst architecture to mimic natural photosynthesis, enabling strong redox potentials and improved charge separation [205]. Nonetheless, photocatalytic carbon dioxide reduction has several limitations, such as low selectivity due to the competing HER, poor carbon dioxide solubility in aqueous media limiting reaction rates, photocatalyst instability under prolonged sunlight exposure and in reactive environments, and complex multi-electron pathways that require precise control of intermediate species, and difficult product separation in mixed gas–liquid systems.

### 5.6. Integration with Electrochemical Storage Systems

Integrating light-driven energy conversion technologies with electrochemical storage systems is essential for creating stable and scalable renewable energy facilities. While solar fuel pathways store energy in chemical bonds, electrochemical storage, primarily batteries and supercapacitors, offers fast response times, high efficiency, and modularity. Combining these systems enables continuous operation, grid stability, and efficient use of intermittent solar resources. Solar-driven devices such as PEC cells, photocatalytic reactors, and artificial photosynthesis systems produce energy intermittently. Electrochemical storage provides temporal smoothing of fluctuating solar output, load balancing for grid-connected systems, energy buffering for off-grid and remote location installations, and hybrid operation where excess solar energy is stored and later used to drive catalytic reactions and power electronics. This synergistic route enhances overall system efficiency and reliability.

#### 5.6.1. Coupling of PV/PEC Devices with Batteries

Solar cells and PEC electrodes can be electrically connected to rechargeable batteries such as lithium-ion for high energy density, sodium-ion and potassium-ion batteries for cost-effective, large-scale storage, flow batteries (vanadium, organic, zinc–bromine) for long-duration storage, and metal–air batteries like zinc–air and lithium–air for high theoretical capacities. Advantages include simple architecture, high round-trip efficiency, and suitability for distributed energy systems. Nonetheless, the associated challenges include the voltage matching between the solar device and the battery, charging electronics, and the degradation due to a fluctuating current.

#### 5.6.2. Solar-Charging of Electrochemical Reactors

Solar energy can directly power electrochemical reactors for water electrolysis leading to hydrogen production, carbon dioxide electroreduction, and nitrogen reduction to ammonia. This hybrid PV–electrolyzer approach is already commercially viable. Benefits include high efficiency using state-of-the-art PV modules, modularity and scalability, and the independent optimization of PV and electrochemical components.

#### 5.6.3. Integrated Photo-Batteries Systems

Photo-batteries combine light absorption and energy storage in a single device. Types of photo-batteries include photo-rechargeable lithium-ion batteries using photoactive cathodes [206], solar-driven redox flow batteries where light directly regenerates redox species [207], and perovskite-based photo-batteries integrating PV layers with solid-state storage [208]. In this latest work, the authors presented an integrated photorechargeable system that combines perovskite solar cells with a solid-state zinc-ion hybrid capacitor, fabricated through a unified process.

**Figure 9:**
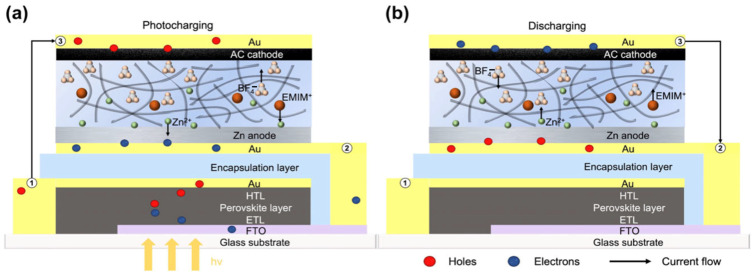
Working principles of the integrated photorechargeable system: (**a**) in photocharging and (**b**) in the discharging process [208].

Central to the design was a UV-curable ionogel electrolyte that effectively shielded the perovskite layer from moisture-induced degradation, allowing the solar cells to operate at their maximum power-conversion efficiency while enabling a monolithic architecture with minimal energy loss. By carefully matching the voltages of the two components and exploiting the high energy-storage efficiency of the hybrid capacitor, the resulting integrated device delivers an overall efficiency of approximately 10% along with enhanced cycling stability. Figure 9 shows the working principles of the integrated photorechargeable system.

Advantages include a compact, multifunctional design, reduced conversion losses, and potential for flexible and wearable applications. Regarding their main limitations, photo-batteries currently face lower efficiency, materials integration constraints, and stability difficulties during cycling and sunlight exposure, making them less mature than separate PV and battery systems [209].

#### 5.6.4. Solar-Driven Supercapacitor Charging

Supercapacitors offer high power density, rapid charge/discharge, and long cycle life. They are ideal for pairing with photodetectors, portable solar devices, and intermittent photocatalytic systems. Hybrid perovskite–graphene photo-supercapacitors are an emerging research area. Hybrid systems that integrate perovskite solar cells with graphene supercapacitors can achieve both photovoltaic conversion and high-power electrochemical storage within a single architecture. These platforms use common carbon electrodes to couple PSCs with graphene supercapacitors, enabling efficient energy transfer and a compact device design. They are being explored to meet the growing demand for miniaturized and multifunctional renewable energy systems. The main advantages are the high energy density provided by the perovskite photo-active layers, the cycling stability from the graphene supercapacitors, and simultaneous solar energy harvesting and storage.

As an elucidative example, a supercapacitor was developed by Ojeda et al. [210] using a photosensitive electrode made from LaFe_0_._94_V_0_._01_Mn_0_._05_O_3_ (V-LFO), a perovskite material that generates photocarriers under sunlight and stores charge through redox reactions. When V-LFO powder was coated onto graphene electrodes, the resulting graphene/V-LFO-supercapacitor showed a capacitance of 581.8 F.g^−1^ and an energy density of 80.8 W.h.kg^−1^ in the dark. Upon exposure to sunlight, the capacitance increased to 876.9 F.g^−1^ and the energy density to 121.8 W.h.kg^−1^, representing approximately a 50% enhancement. This improvement arises from the additional photogenerated electrons and holes in the graphene/V-LFO composite, which directly contribute to the redox-based charge storage process. Because the V-LFO electrode is in contact with an aqueous electrolyte, illumination also triggers the photocatalytic formation of O_2_^2−^ and OH radicals. These negatively charged species migrate toward the positive electrode, where they are stored via EDL mechanisms or react with the V-LFO material to create new compounds and oxygen vacancies, further contributing to the redox-driven energy storage. 

Table 10 summarizes the characteristics and roles of these electrochemical storage systems in solar systems.

## 6. Manufacturing and Scalability

### 6.1. Manufacturing Techniques

Manufacturing is the bridge between laboratory optoelectronic materials and real-world renewable-energy devices. The choice of fabrication technique determines not only the performance but also the scalability, cost, environmental impact, and long-term reliability. Solution processing [211], vapor deposition [212], and printing [213] are the main optoelectronic manufacturing techniques. Each technique exhibits distinct advantages and limitations depending on the material system and application.

#### 6.1.1. Solution Processing

Solution processing enables low-temperature and cost-effective fabrication of thin films from liquid inks and precursor solutions. It is essential for perovskites, organic semiconductors, colloidal QDs, and some metal oxide systems. This technique is compatible with flexible substrates, scalable to roll-to-roll manufacturing, and enables rapid prototyping and composition adjustment. Solution processing techniques include spin coating, which enables the production of uniform thin films for laboratory-scale devices; dip coating, which provides large-area uniformity for coatings and electrodes, sol–gel processing for oxide films with tunable porosity, and spray pyrolysis that enables the scalable deposition of oxides and catalysts. The advantages of solution processing are low energy consumption, suitability for emerging materials like perovskites, organic semiconductors, and QDs, and elevated throughput in industrial scenarios. The main limitations are that solvent control affects film uniformity, sensitivity to humidity and temperature, and reproducibility challenges on a large scale.

#### 6.1.2. Vapor Deposition

Vapor-phase methods produce highly uniform, dense, and stable films, which are ideal for high-performance photovoltaics and optoelectronics. They offer high purity and fine thickness control, suitable for inorganic semiconductors and multilayer stacks, and enable conformal coatings on complex geometries. The vapor deposition techniques include Physical Vapor Deposition (PVD)—evaporation, sputtering; CVD for TMDs, graphene, silicon, and III–V materials; ALD for ultrathin, pinhole-free layers for passivation; and metal–organic CVD (MOCVD), which is most suitable for III–V multijunction solar cells. The corresponding beneficial features include superior film quality and stability, fine control of composition, and suitability for tandem and multijunction configurations. However, limitations include high capital and operating costs, limitations imposed by vacuum systems on throughput, and unsuitability for flexible substrates.

#### 6.1.3. Printing Techniques

Printing transforms optoelectronic manufacturing into a high-throughput, additive, and scalable process. It is especially promising for flexible, lightweight, and large-area devices. Additive manufacturing reduces material waste, is compatible with roll-to-roll production, and enables patterned deposition without the need for photolithography. The principal techniques include inkjet printing, used for digital patterning of perovskites, organic semiconductors, and electrodes; screen printing, widely used for silicon solar-cell metallization; gravure and flexographic printing—high-speed roll-to-roll fabrication; and aerosol-jet printing—fine-feature deposition for interconnects. The advantages of these techniques are low cost and high throughput, making them ideal for flexible PV, sensors, and wearable devices, while enabling multi-material integration. The main limitations are associated with ink formulation complexity, lower resolution compared to photolithography, film uniformity, and defect control. Table 11 summarizes the benefits, limitations, and applications of the main optoelectronic manufacturing techniques.

**Figure 10:**
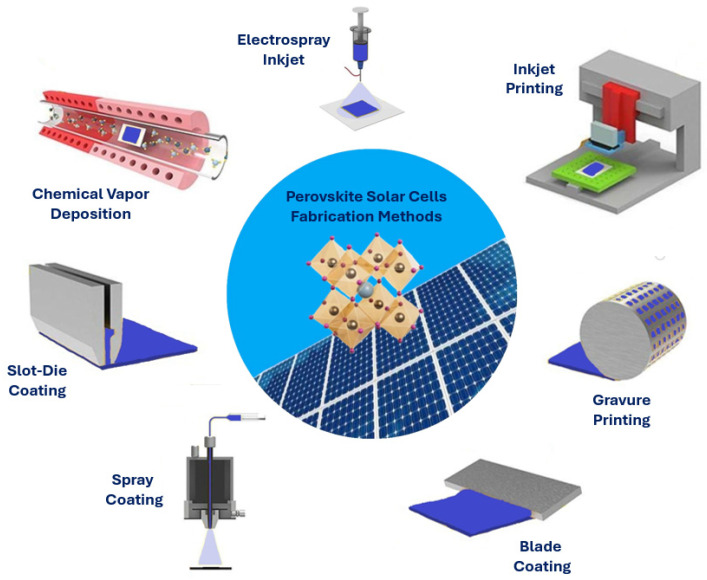
Main manufacturing methods for perovskite solar cells.

These techniques matter for the scalability and reliability of optoelectronic devices. Scalability depends on throughput, material cost, and compatibility with large-area substrates. In contrast, reliability is strongly influenced by film uniformity, defect density, and interfacial quality, which are areas where vapor deposition excels, but printing and solution processing are rapidly improving. Also, hybrid manufacturing techniques, such as a combination of vapor-deposited transport layers and solution-processed absorbers, are becoming the dominant policy for perovskite and tandem PV. Furthermore, in the case of perovskite solar cells, Figure 10 shows the fundamental manufacturing processes for this type of cell.

Blade coating, gravure printing, slot-die coating, spray coating, inkjet printing, CVD, and electrospray inkjet are all established or emerging fabrication methods used to deposit perovskite layers and other functional films in perovskite solar cell manufacturing. Blade coating is a simple, low-cost, solution-based technique where a blade spreads precursor ink across a substrate, making it suitable for large-area and roll-to-roll processing. Also, gravure printing uses engraved rollers to transfer perovskite inks with high throughput and scalability, though it requires precise ink rheology control. Slot-die coating dispenses a precursor solution through a narrow slit to form uniform films with minimal material waste, making it one of the leading scalable deposition methods for perovskite modules. Moreover, spray coating technique atomizes precursor solutions into fine droplets that deposit onto the substrate, enabling fast, large-area coverage but requiring careful control to achieve uniform thickness. Inkjet printing digitally deposits patterned droplets of a perovskite precursor, offering maskless patterning and low waste, though challenges such as coffee-ring effects must be managed.

In contrast, CVD is a vapor-phase technique that forms high-quality, pinhole-free perovskite films with enhanced uniformity and stability, though it is more complex and costly than solution processing. In addition, electrospray inkjet uses an electric field to generate ultra-fine charged droplets, enabling controlled crystallization and nanostructured coatings, though with lower throughput than other scalable methods. Together, these techniques span the solution-processed and vapor-processed approaches, supporting both laboratory-scale optimization and large-scale manufacturing of perovskite solar cells.

A key scalability trade-off emerges between solution processing and vapor deposition, especially for perovskite and tandem cell manufacturing. Solution processing enables low-temperature and cost-effective fabrication of thin films from liquid inks and precursor solutions and is compatible with flexible substrates, scalable to roll-to-roll manufacturing, and enables rapid prototyping and composition adjustment, making it attractive for high-throughput, large-area perovskite layers and low CAPEX expansion. However, when scaled to modules with square meters of area, solution and printing procedures present high sensitivity to ink rheology, humidity, and web speed, which can promote pinholes, grain-boundary networks, and thickness variations, leading to shunts, hotspots, and failures at the module scale.

By contrast, vapor deposition offers uniform, dense, and stable films, which are very suitable for high-performance photovoltaics and optoelectronics, with improved thickness control and conformal coverage that benefit tandem and multijunction architectures where interfacial quality and defect suppression are critical. However, these advantages entail high capital investment, vacuum-limited throughput, and limited compatibility with flexible substrates, which can constrain gigawatt-scale deployment of perovskite and tandem technologies compared to incumbent silicon lines. As a result, hybrid manufacturing, which combines vapor-deposited transport layers and solution-processed absorbers, is emerging as a pragmatic compromise, leveraging the uniformity and reliability of vapor-deposited stacks for critical interfaces while retaining the low-cost, roll-to-roll potential of solution-processed perovskite absorbers for scalable tandem integration.

### 6.2. Scaling Challenges and Reliability Testing

Scaling optoelectronic renewable-energy technologies from laboratory devices to bankable modules is governed by two interdependent factors: manufacturing scalability and demonstrated reliability under standardized tests. Even when small-area cells achieve high efficiencies, module-scale fabrication introduces extra failure characteristics like non-uniform coatings, interfacial defects, and encapsulation weaknesses, which affect the yield, durability, and long-term performance.

#### 6.2.1. Lab-to-Module Scaling

Defects that are tolerable at the millimeter scale, such as pinholes, grain-boundary networks, and thickness variations, become critical failure points when extended across square-meter modules. These imperfections can produce shunts, hotspots, and early-life failures. As a result, laboratory deposition methods must be translated into tight industrial process windows, where temperature, humidity, ink rheology, web speed, and drying/annealing conditions are controlled with precision.

Large-area coating methods including slot-die, blade coating, spray deposition, and roll-to-roll printing, require uniform wet-film formation and defect-free drying across wide substrates. On the other hand, hybrid manufacturing routes, such as combining vapor-deposited transport layers with solution-processed absorbers, must integrate seamlessly with existing silicon and thin-film module lines without imposing excessive capital expenditure. Therefore, achieving reproducibility at scale depends on robust metrology, inline monitoring, and process-control procedures capable of detecting defects before lamination.

#### 6.2.2. Industrial Reliability Testing

Module-level reliability is assessed through standardized accelerated-stress protocols, most prominently IEC 61215 [92], which incorporates tests required for bankability and grid connection. These tests evaluate the ability of a module to withstand prolonged periods of environmental exposure by applying controlled thermal, humidity, mechanical, and radiative stress.

The damp heat (DH) test—typically 1000 h at 85 °C and 85% relative humidity—probes moisture ingress, corrosion, and encapsulant stability [214]. Recent climate-mapping studies show that the equivalent of a 30-year field exposure varies significantly by region, with DH equivalence ranging from 750 to 2250 h depending on local temperature–humidity profiles and the activation energy of the dominant degradation pathway [215]. This emphasizes the need to interpret the results from the DH test according to the specific deployment climate rather than as an absolute lifetime metric.

The thermal-cycling (TC) test subjects modules to repeated transitions between low and high temperatures, mimicking diurnal and seasonal swings. The TC test interacts with Light-Induced Degradation (LID) and light- and elevated-temperature-induced degradation (LETID) in silicon modules, which can complicate the interpretation and produce false passes or failures if not properly decoupled [216]. Additional IEC tests, including humidity–freeze, UV exposure [217], and mechanical loading, evaluate delamination [218], microcracking, and encapsulant/photoactive-layer degradation [219] under combined environmental and mechanical stress.

#### 6.2.3. Encapsulation Performance and Moisture Ingress

Encapsulation is a primary determinant of module-level reliability. Moisture diffusion through polymer encapsulants during DH testing can range from 0.5–2 to 5 g m^−2^ day^−1^, with interfaces and edge seals acting as dominant ingress pathways. Moisture ingress accelerates corrosion, delamination, and interlayer chemical reactions, making encapsulation design based on material selection, edge-seal architecture, and lamination conditions central to long-term durability.

Because encapsulation failures often originate from microscopic defects introduced during manufacturing, monitoring using optical, electrical, and X-ray tools is essential for detecting voids, adhesion defects, and delamination precursors before the final lamination. It is now widely considered best practice that reliability should be included in the stack design from the beginning by considering DH, TC, and UV stresses early, rather than treating reliability as an issue to check only at the end.

#### 6.2.4. Linking Accelerated Tests to Real-World Performance

A critical challenge in module qualification is translating accelerated-test results to real-world climates. Models that couple activation energies, local meteorological data, and stress-accumulation functions are needed to avoid both under-testing, which leads to premature field failures and over-testing, which leads to unnecessary costs and conservative warranties. Such models also refine warranty structures and Levelized Cost of Electricity (LCOE) projections by providing more accurate lifetime estimates.

### 6.3. Cost, Scalability, and Lifecycle Analysis

#### 6.3.1. Cost and Learning Curves

For PV and related technologies, LCOE folds combine CAPEX, OPEX, efficiency, degradation rate, and lifetime. Recent system-dynamics work shows how technological learning—through higher efficiencies, thinner wafers, better yields, and cheaper balance-of-system components—drives down LCOE as cumulative installed capacity grows. Moreover, PV has shown learning rates of around 20% or more, meaning each doubling of cumulative capacity cuts module prices by approximately one-fifth. This relationship is known as Swanson’s Law and is supported by multiple empirical datasets. Additionally, the updated International Technology Roadmap for Photovoltaics (ITRPV) roadmaps link this to specific process improvements like wafer thinning, high-efficiency cell architectures, module integration, and project continued LCOE reductions as manufacturing scales further.

A recent review [220] emphasizes that Life-Cycle Cost Analysis (LCCA), combining economic and technical parameters over the complete lifetime, is now standard for PV project evaluation, especially when comparing emerging technologies (perovskites, tandems) with the incumbent crystalline silicon [221].

#### 6.3.2. Scalability Constraints

High-volume PV manufacturing is currently dominated by technologies capable of sustaining gigawatt-scale throughput with high yields, notably crystalline silicon and CdTe. Emerging solution-processed and printed technologies must demonstrate comparable scalability without compromising uniformity, reliability, or device performance. Scalability potential is also constrained by the availability of critical materials such as indium, gallium, tellurium, and silver, and by precursor purity requirements. Learning curve analyses increasingly incorporate reductions in material intensity, such as thinner wafers and lower silver consumption, as major drivers of future cost reductions [222]. In terms of standardization and bankability, large-scale deployment requires compliance with established reliability standards and the accumulation of long-term field performance data. Without this validation, even technologies that combine low cost and high efficiency are unlikely to progress beyond the pre-commercial stage.

#### 6.3.3. Lifecycle and Environmental Assessment

Life cycle assessment (LCA) evaluates environmental impacts across all stages of a photovoltaic system, including raw material extraction, manufacturing, transportation, operation, and end-of-life management. Recent studies indicate that most environmental impacts are concentrated in the manufacturing phase, while operational emissions remain close to zero [223]. Advanced methodologies now integrate learning curves with LCA to project how environmental footprints, such as carbon dioxide-equivalent emissions per kWh, evolve as technology matures and decarbonizes [224]. In this context, environmental learning rates can be applied to parameters such as energy and material consumption to forecast further sustainability improvements. There is growing consensus that Life Cycle Cost Analysis (LCCA) should be coupled with LCA, enabling decision-making that simultaneously accounts for economic and environmental performance. This approach is particularly relevant when comparing silicon-based technologies with emerging solutions such as perovskites, tandem cells, and hybrid systems.

As PV deployment expands, end-of-life management becomes increasingly important. Indeed, recycling processes already recover materials such as glass, aluminum, and silicon, while advanced strategies target elements like silver and indium. On the other hand, the prospective LCA studies show that higher recycling rates and the use of low-carbon electricity in manufacturing can considerably reduce environmental impacts and decrease the Levelized Cost of Electricity (LCOE) via material recovery.

## 7. Machine Learning

Machine learning (ML) is emerging as one of the most transformative tools in optoelectronics and renewable energy research. While this review has highlighted the advances in materials science, device architectures, and light–matter interactions, ML acts as an enabling framework that accelerates progress across all these domains. 

### 7.1. Machine Learning Optimization of Optoelectronic Devices

Machine learning is increasingly used to design and optimize optoelectronic device architectures by enabling the fast exploration of large structural, optical, and electronic parameter spaces that are impractical to investigate using conventional experimental or simulation approaches. By learning structure–property relationships, ML significantly accelerates the development of high-performance photovoltaic, photoelectrochemical, and photonic systems. High-fidelity simulation methods such as Finite-Difference Time-Domain (FDTD) [225], drift–diffusion modeling, and density functional theory offer accurate predictions but are computationally intensive. ML surrogate models can approximate these simulations with orders-of-magnitude reductions in computational cost. These models enable essential capabilities, including predicting the absorption spectra for multilayer stacks and nanostructured surfaces, estimating the current–voltage characteristics under varying sunlight intensity, temperature, and defect conditions, and modeling charge carrier transport in complex geometries such as textured interfaces or graded bandgap structures.

**Figure 11:**
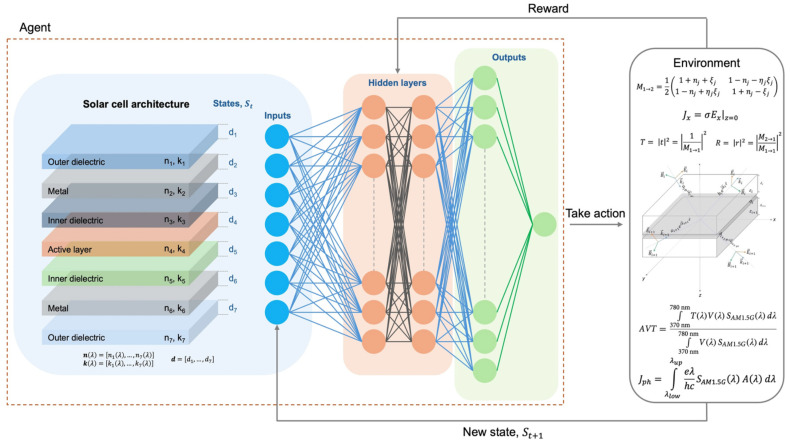
Neural network structure of the deep Q-learning model for determining the optimal semi-transparent organic solar cells architecture [228].

These surrogate models allow rapid iteration over thousands of device configurations, guiding researchers toward the best configuration. Device performance is highly sensitive to nanoscale structural parameters. ML optimization frameworks, often combining neural networks with evolutionary algorithms and Bayesian optimization, can efficiently navigate these design spaces. Principal applications include deep learning for tandem solar cells, optimizing top-cell bandgap, interlayer thickness, and recombination layers to maximize current matching [226], and organic photovoltaics, where donor–acceptor ratios, blend morphology, interfacial energetics, and device architecture are optimized [227,228] using neural network structures such as e deep Q-learning models, as shown in Figure 11.

The nanostructured optical elements, such as metasurfaces, photonic crystals, and plasmonic arrays, enable enhanced light trapping, spectral selectivity, and directional emission. Their vast design space makes them particularly suitable for ML optimization. ML enables inverse design of nanostructures that achieve target absorption or scattering spectra, rapid evaluation of geometric variations, replacement of computationally intensive electromagnetic solvers, and discovery of unconventional geometries that outperform traditional periodic and symmetric designs. These advances support high-efficiency solar cells, photodetectors, and photocatalytic platforms. Additionally, ML models trained on accelerated aging data and thermal simulations can predict hotspot formation in photovoltaic modules [229,230], mechanical stress accumulation in flexible and multilayer devices [231,232], degradation pathways from moisture, UV exposure, and ion migration [233], and failure probabilities under varying environmental conditions, including short circuits, shading, line-to-line faults, and open-circuit events [234].

**Figure 12:**
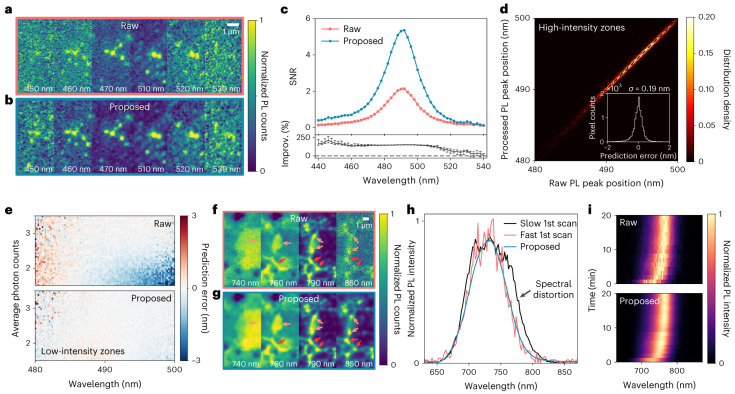
Normalized PL-intensity maps of a self-assembled CsPbBr_3_ perovskite NPL film comparing raw (**a**) with processed (**b**) mapping from 450 to 530 nm, (**c**) SNR (**above**) and percentage improvement (**below**) after image restoration relative to wavelength-specific images, (**d**), Micro-PL peak position estimations based on raw and processed data for regions with high PL intensities. Inset: histogram of differences between raw and processed predictions, (**e**) Peak-wavelength estimation based on the raw data (**above**) and processed data (**below**) against the manually derived ground truth. PL images of triple-cation Cs_0.05_FA_0.78_MA_0.17_Pb(I_0.83_Br_0.17_)_3_ perovskite films comparing raw (**f**) and processed (**g**) normalized PL-intensity maps—the arrows mark local grain regions with different emission, suggesting alterations in halide ratios within these regions, (**h**) Local-PL spectra of thermally evaporated wide-gap FA_0.7_Cs_0.3_Pb(I_0.6_Br_0.4_)_3_ perovskite films of one camera pixel, comparing the PL spectra of the first captured scan with an integration time of 1 s (slow scan) and 0.1 s (fast scan) per wavelength step, (**i**) Local-PL evolution of the wide-gap perovskite film captured by fast scan (**above**) and proposed method (**below**) during twenty minutes [233].

The work [235] introduced a self-supervised machine learning framework that leveraged multi-channel correlation and blind denoising to reconstruct images without the need for high-quality reference data, enabling rapid and low-dose measurements. Using this approach, the authors performed operando luminescence mapping on emerging optoelectronic semiconductors, including organic and halide perovskite photovoltaic and light-emitting devices. By monitoring spatially resolved electroluminescence degradation in mixed-halide perovskite blue LEDs, it was revealed that lateral ion migration, occurring perpendicular to the applied electric field, drove the formation of chloride-rich, defect-dominated regions with suppressed emission. This degradation pathway would remain inaccessible using conventional imaging techniques. Figure 12 shows the application of this method to halide perovskites.

These insights inform the design of more durable architectures and guide improvements in encapsulation, interlayer engineering, and thermal management strategies. Concerning the work performed in [230], the authors stated that one of the most frequent faults in photovoltaic systems is the formation of hot spots on the solar panels caused by cell mismatch, which results from uneven environmental conditions or physical damage. These underperforming cells generate excess heat, creating localized hot spots. Traditionally, hot-spot identification relies on thermal imaging followed by manual image processing and inspection. With the fast advancement of ML image analysis and diagnostic techniques, it has become increasingly feasible to develop intelligent models capable of detecting hot spots with greater accuracy. In this work, the authors introduced a RetinaNet-based approach designed to identify hot spots in photovoltaic panels using thermal images, offering a more robust and scalable solution for solar system monitoring.

**Figure 13:**
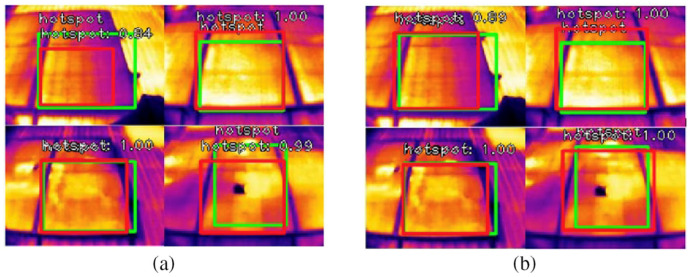
RetinaNet results with group labeling - green bounding boxes represent the ground-truth annotations generated during the labeling process and the red bounding boxes indicate hot-spot regions predicted by the model: (**a**) ResNet-50. (**b**) ResNet-152 [230].

Figure 13 presents the prediction results for a batch of four thermal images from the test set using RetinaNet models with ResNet-50 and ResNet-152 backbones under two different labeling procedures. In each image, the green bounding boxes represent the ground-truth annotations generated during the labeling process, while the red bounding boxes indicate hot-spot regions predicted by the model. Both box types are accompanied by the object class label “hotspot,” and the predicted boxes display the confidence score produced by the classification subnet.

**Figure 14:**
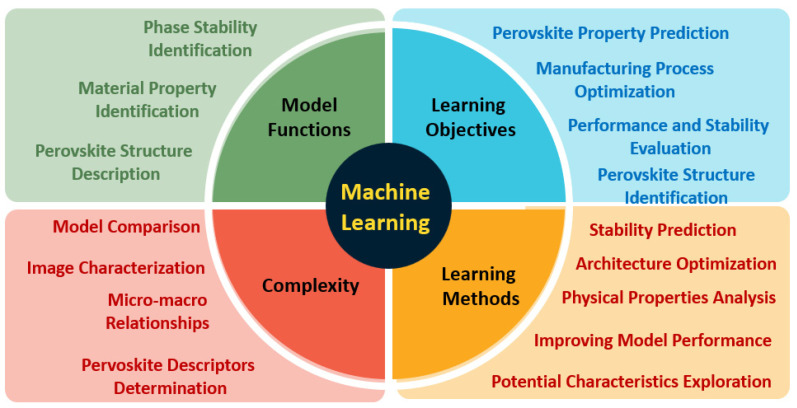
ML model functions, and learning objectives and methods for improving perovskite solar cells.

ML is also increasingly integrated into fabrication workflows, enabling the real-time optimization of device configurations. Examples are the adaptive control of material deposition parameters to achieve optimal film thickness and morphology, enhancement of interface quality, and defect detection [235]. This approach is particularly impactful for scalable manufacturing techniques such as roll-to-roll coating, vapor deposition, and inkjet printing. Figure 14 summarizes the functions of ML models, as well as their learning objectives and methods for improving perovskite solar cells.

### 7.2. Machine Learning Optimization of Optoelectronic Materials

AI and ML are emerging as powerful tools to accelerate the discovery, optimization, and deployment of optoelectronic materials for renewable energy applications. Their integration addresses key bottlenecks in traditional trial-and-error approaches, enabling faster innovation cycles. The main AI and ML applications in this field are the following:

- High-throughput screening and property prediction—ML models, such as graph neural networks and random forests, are used to predict key material properties (bandgap, stability, carrier mobility, defect formation energy) from compositional and structural descriptors, significantly reducing the need for expensive DFT calculations [236].
- Process optimization and manufacturing control—ML algorithms optimize fabrication parameters, such as annealing temperature, precursor ratios, and spraying conditions, to improve film uniformity, crystallinity, and yield. Techniques such as ALS spraying have benefited from data-driven procedures to achieve better thickness and bandgap control [80].
- Device performance prediction and degradation modeling—Convolutional neural networks (CNNs) and physics-informed neural networks are employed to predict efficiency, identify hotspots, and model long-term degradation under real operating conditions.
- Autonomous self-driving laboratories—Closed-loop systems integrating robotics, in situ characterization, and active learning enable fully autonomous experimentation, dramatically accelerating the discovery of stable and efficient optoelectronic materials.

These AI-driven strategies are particularly impactful for complex hybrid and nanostructured systems, where multidimensional parameter spaces make traditional optimization impractical. By combining experimental data with computational modeling, ML not only accelerates materials discovery but also supports smart integration and real-time performance optimization in renewable energy systems.

### 7.3. Machine-Learning of Light–Matter Interactions

Light–matter interactions lie at the core of all optoelectronic technologies, governing absorption, emission, charge generation, and catalytic activity. Traditionally, optimizing these interactions requires computationally demanding electromagnetic simulations or labor-intensive experimental tuning. ML is transforming this landscape by providing fast, flexible, highly expressive tools for modeling, predicting, and designing optical responses across complex material and structural spaces. These capabilities hasten the development of solar cells, photodetectors, photocatalysts, and nanophotonic systems with unprecedented performance. However, electromagnetic solvers such as Finite-Difference Time-Domain (FDTD) and the Finite Element method (FEM) are accurate but computationally expensive, particularly for three-dimensional nanostructures and broadband simulations [237]. ML surrogate models trained on simulation datasets can approximate optical responses with near-instantaneous inference. Advantages include fast prediction of absorption, reflection, and scattering spectra for multilayer and nanostructured systems, efficient modeling of near-field enhancements in plasmonic and dielectric nanostructure; and real-time exploration of geometric parameter spaces, enabling interactive design workflows [238,239,240].

These surrogate models reduce computational costs by several orders of magnitude, making large-scale optimization feasible. Inverse design, in which an optical response is specified, and the algorithm proposes a corresponding structure, is one of the most impactful applications of ML in photonics. This includes neural network-based design of metasurfaces [241], photonic crystals [242], and plasmonic arrays [243], as well as hybrid physics-informed ML frameworks that enforce Maxwell’s equations while exploring unconventional design spaces [244]. These approaches have enabled the discovery of nanostructures with enhanced light trapping, angle-insensitive absorption, and tailored spectral selectivity for photovoltaic and photocatalytic applications. ML also accelerates the identification of structures that maximize absorption while minimizing parasitic losses [245].

**Figure 15:**
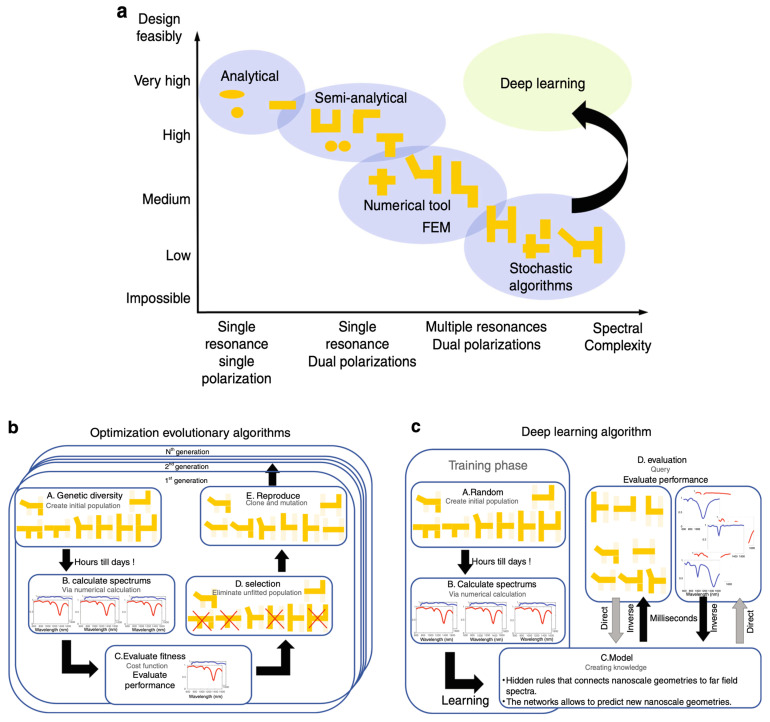
(**a**) Current computational tools are efficient for direct modeling, predicting the optical response of a nanostructure from its geometry and materials—whereas the more design-relevant inverse problem of generating a structure for a response remains slow and inefficient. (**b**) In a Genetic Algorithm workflow, an initial population of diverse nanostructures is created, spectra are computed, fitness is evaluated, low-fitness candidates are removed, and the best structures are cloned and mutated until the optimal design is found. (**c**) Building a Deep Learning model involves assembling a diverse dataset, calculating spectra, training a network to learn the underlying optical rules, and then querying it with new target responses; effective generalization requires the model to internalize the physics governing the nanophotonic behavior [245].

This latest work experimentally demonstrated that a deep neural network trained on thousands of synthetic experiments can not only extract subwavelength structural parameters from far-field measurements alone but also directly solve the inverse design problem. This capability enables the design and characterization of metasurface-based optical components, as well as the optimization of nanostructures tailored for specific chemical and biomolecular applications. Figure 15 shows the adopted methodology.

**Figure 16:**
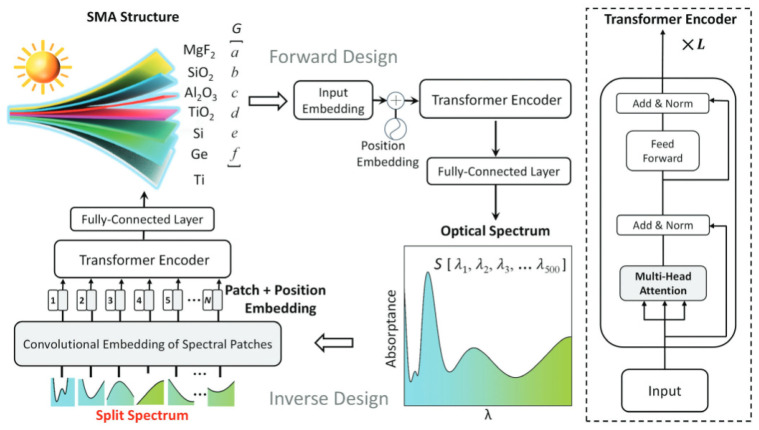
MST architecture for the smart design of graded-refractive-index solar metamaterial absorbers [247].

Principal applications include textured interfaces optimized for broadband absorption in silicon tandems, disordered and quasi-random nanostructures that outperform periodic designs [246], plasmonic and Mie-resonant nanoparticles for hot carrier generation, multiscale architectures combining micro- and nanoscale features for synergistic light management, and investigations on broadband solar metamaterial absorbers [247]. This latest study introduced a deep-learning framework based on a Metamaterial Spectrum Transformer (MST) that enables a high-performance solar metamaterial absorber design. By partitioning the optical spectrum into N patches, the MST effectively mitigates overfitting issues common in conventional deep-learning models and substantially enhances learning capability. Building on this architecture, the authors developed a flexible, user-defined design platform that supported real-time, on-demand creation of metamaterials with diverse optical functionalities. They further applied this methodology to design and fabricate solar metamaterial absorbers with graded-refractive-index nanostructures, achieving an average absorptance of 94% across the broadband solar spectrum. Also, outdoor testing indicated an annual solar-energy collection of approximately 1061 kWh m^−2^, emphasizing the high efficiency of the system. Figure 16 illustrates the MST network consisting of inverse design and forward design. 

ML-designed systems often achieve performance levels beyond those attainable through conventional intuition-driven approaches [248,249]. Moreover, many emerging optoelectronic platforms rely on complex quantum and collective excitations. ML provides the most suitable tools to model these interactions, including predicting exciton binding energies in low-dimensional semiconductors, modeling plasmon–exciton coupling in hybrid nanostructures, designing of strong-coupling cavities for enhanced photocatalysis and nonlinear optics, and learning potential energy surfaces for photoinduced charge transfer. These models help the research community design systems where quantum effects enhance solar conversion and catalytic efficiency. ML also accelerates the design of photocatalysts and PEC interfaces by linking optical properties to catalytic performance. Capabilities include predicting absorption edges and charge transfer efficiencies, optimizing plasmon resonances to enhance local electromagnetic fields and reaction rates, designing nanostructured catalyst supports, and correlating illumination conditions with reaction kinetics using data-driven models. This is particularly impactful for carbon dioxide reduction, water splitting, and nitrogen fixation. Physics-informed neural networks (PINNs) incorporate Maxwell’s equations, material dispersion relations, and boundary conditions into the learning process. Their advantageous features include improved accuracy with limited training data, enhanced generalization across wavelengths and geometries, the ability to solve simultaneously forward and inverse problems, and reduced reliance on large simulation datasets. As a result, PINNs are emerging as a very promising framework for designing optoelectronic systems, while ensuring physical consistency [250].

### 7.4. Machine-Learning in Solar Chemical Conversion

The solar-driven chemical conversion that relies on Photoelectrochemical (PEC) water splitting, carbon dioxide reduction, nitrogen fixation, and solar-assisted fuel synthesis manages a complex interplay of optical absorption, charge separation, interfacial charge transfer, and catalytic surface reactions. These processes span multiple length and time scales, making them difficult to optimize by traditional trial-and-error experimentation. In this context, ML has emerged as a powerful tool to expedite catalyst discovery, interface modification, reaction pathway modeling, and system-level optimization, enabling the implementation of more efficient and durable solar chemical energy systems. Identifying active, selective, and stable catalysts for PEC and photocatalytic reactions remains a central challenge.

ML models trained on experimental datasets, Density Functional Theory (DFT) calculations, and high-throughput screening results can rapidly predict catalytic properties. Their capabilities include predicting adsorption energies of fundamental intermediates in water splitting, carbon dioxide reduction, and nitrogen reduction reactions, identifying active sites on complex surfaces, including defects, dopants, and heterostructure interfaces, screening catalyst compositions such as oxides, sulfides, nitrides, and MXenes, and discovering multicomponent systems like high-entropy alloys and doped oxides with optimized reaction kinetics. All these capabilities considerably reduce the time and cost needed to pinpoint the most promising catalytic materials. In addition, since solar chemical conversion depends on charge transfer across semiconductor–electrolyte interfaces, ML provides new tools to model and optimize these processes. Principal applications are predicting charge transfer rate constants from optical, electrochemical, and spectroscopic data, learning structure–reactivity relationships for surface terminations, passivation layers, and co-catalysts, modeling interfacial band alignment and its evolution under illumination and applied bias, and identifying degradation routes such as photocorrosion, ion migration, and catalyst poisoning. These insights guide the design of interfaces that minimize recombination losses and maximize catalytic turnover. Furthermore, ML accelerates the design of nanostructured photocatalysts by interlinking geometry, composition, and optoelectronic properties.

Examples include inverse design of nanostructured semiconductor surfaces for enhanced light harvesting [251], optimization of plasmonic–semiconductor hybrids to exploit hot-carrier generation, the design of hierarchical architectures combining micro- and nanoscale features for improved mass transport and photon utilization, and the prediction of optimal catalyst loading and spatial distribution on PEC electrodes [252,253,254]. Notably, these approaches often reveal non-intuitive structures that outperform conventional designs. ML models trained in DFT data or experimental kinetics can also map complex reaction networks. This enables the prediction of reaction intermediates and rate-limiting steps, identification of competing pathways that reduce selectivity, learning free-energy landscapes for multi-electron/multi-proton reactions, and correlation between catalyst structure and product distribution, which is relevant for carbon dioxide reduction. These mechanistic insights support catalyst design and process optimization. Beyond materials design, ML is increasingly used to optimize operating conditions and the performance of solar chemical reactors. Principal applications include predicting optimal sunlight profiles (e.g., spectral tuning and pulsed operation), optimizing electrolyte composition, pH, and flow conditions, modeling mass transport limitations, and designing PEC cell configurations that maximize photon utilization while minimizing resistive losses [255].

These tools help bridge the gap between laboratory experiments and solar fuel production. Finally, self-driving laboratories are beginning to transform solar chemical research by integrating ML with automated synthesis, characterization, and testing [256]. These systems enable automated catalyst synthesis guided by ML-predicted compositions, real-time optimization of reaction conditions based on performance feedback, and closed-loop discovery cycles that rapidly converge toward high-performance materials and configurations.

### 7.5. Machine Learning Smart-Grid Integration and Energy System Optimization

As optoelectronic technology matures and scales, its impact increasingly depends on how it can be integrated into broader energy systems. Solar photovoltaics, solar-driven chemical reactors, and distributed optoelectronic assets introduce variability, intermittency, and complex spatiotemporal dynamics into the grid. ML provides powerful tools to forecast generation, optimize storage, coordinate distributed resources, and enhance grid stability—capabilities that are essential for transitioning toward resilient, intelligent, and integrated renewable energy systems. Accurate forecasting of solar generation is critical for grid balancing, dispatch planning, and storage management. ML models outperform traditional physical approaches by learning nonlinear relationships across diverse datasets.

The main contributions include short-term irradiance forecasting [257] using satellite imagery, sky cameras, and meteorological data, long-term generation prediction incorporating seasonal variability, degradation trends, and environmental factors, spatiotemporal forecasting for distributed PV systems and solar farms [258,259], and hybrid physics–ML models that combine radiative transfer principles with data-driven corrections [260]. These capabilities reduce margins and improve reliability. In addition, energy storage systems that rely on batteries, hydrogen, and thermal reservoirs play a relevant role in mitigating issues associated with solar intermittency. ML enables the coordination of these assets to maximize efficiency and minimize costs. Principal applications include the predictive control of battery charging/discharging based on forecasted supply and demand, optimization of hybrid storage systems (e.g., battery–hydrogen or battery–thermal), state-of-health prediction for batteries and electrolyzers, enabling predictive maintenance, and load-shifting strategies that align consumption with solar availability.

Modern power grids increasingly rely on Distributed Energy Resources (DERs), including rooftop PV, community solar installations, microinverters, electric vehicle chargers, and residential energy systems. ML enables real-time coordination of these heterogeneous assets. Capabilities include multi-agent reinforcement learning for decentralized DER coordination, dynamic inverter control for voltage and frequency regulation, the aggregation of DERs into Virtual Power Plants (VPPs), and detection of anomalies across distributed networks. This distributed intelligence enhances system resilience while reducing the need for large-scale infrastructure upgrades. Furthermore, solar chemical systems—such as hydrogen production, CO_2_-to-fuel conversion, and ammonia synthesis—can serve as flexible loads and energy storage pathways. ML optimizes their integration within the broader energy ecosystem. Principal applications include scheduling hydrogen production during periods of excess solar generation, predicting catalyst degradation to enable maintenance planning, optimizing electrolyzer operation for grid support services, and the co-optimization of electricity and fuel markets using data-driven economic models.

Moreover, ML models trained on grid sensor data (e.g., PMUs, SCADA systems, and smart meters) can detect anomalies and predict failures. Principal applications include the detection of voltage instabilities, prediction of transformer and inverter failures, and real-time grid reconfiguration to isolate faults and maintain service continuity. Finally, digital twins, which are virtual replicas of solar farms, microgrids, and distribution networks, are increasingly used for optimization and control. ML enhances these systems through adaptive, data-driven modeling. The benefits include simulation of grid behavior under different renewable penetration scenarios, optimization of dispatch strategies, real-time synchronization with sensor data for predictive control, and evaluation of infrastructure upgrades prior to physical deployment.

### 7.6. Machine Learning and Data-Driven Design in Perovskite and Hybrid Photovoltaics

ML has become a central driver of perovskite photovoltaic research by enabling predictive modeling of composition–property relationships, defect chemistry, ion migration, and long-term degradation. Also, models trained on defect formation energies and migration barriers can pinpoint compositions with lower halide mobility, offering a data-driven way to reduce hysteresis and improve stability. Neural networks and Gaussian process regressors can map precursor chemistry, solvent environment, and annealing conditions to microstructural characteristics such as grain size, orientation, and defect density, allowing predictive control of film formation. Moreover, ML-accelerated DFT workflows further support the screening of transport layers by predicting band alignment, interfacial dipoles, and recombination-relevant energetics.

Beyond materials prediction, self-supervised imaging frameworks reconstruct operando luminescence maps without high-quality reference datasets, revealing degradation pathways, including lateral ion migration and defect clustering, that conventional imaging cannot resolve. On the other hand, supervised learning remains dominant for forecasting device metrics like PCE, VOC, JSC, fill factor, and stability from compositional and processing descriptors, while convolutional and physics-informed neural networks enable hotspot detection, multispectral image fusion, and mechanistic degradation modeling. Time-series architectures such as LSTMs and temporal convolutional networks predict performance decay under thermal, humidity, and illumination stress and detect early-stage degradation signatures long before device failure. In manufacturing contexts, ML supports defect detection, optimization of scalable coating methods, predictive maintenance of deposition and annealing tools, and yield forecasting in pilot-scale module production. Moreover, emerging reinforcement-learning platforms are enabling autonomous laboratories that optimize deposition and annealing conditions, while physics-informed ML frameworks integrate semiconductor physics and defect chemistry to improve interpretability, reduce data requirements, and enhance model generalizability.

Despite these advances, progress is limited by data scarcity, non-standardized protocols, limited model transferability across the perovskite families, and the difficulty of embedding physical constraints into purely data-driven architectures. Overcoming these limitations will need standardized datasets, hybrid physical-ML models, and tighter integration between computational predictions and automated experimental workflows.

## 8. Commercialization and Policy

Emerging photovoltaic and optoelectronic energy technologies, such as perovskites, tandem cells, organic PV, and advanced PEC systems, are rapidly transitioning from laboratory research toward early-stage deployment. However, the path to full commercialization is shaped by technical bottlenecks, manufacturing challenges, and policy frameworks that will determine large-scale adoption. Material instability and reliability remain critical issues. Many emerging PV materials are still vulnerable to moisture, oxygen, UV radiation, and thermal stress. Considering the existing standardized protocols, passing the IEC 61215/61730 [92,261] certification is necessary but insufficient, as financier stakeholders require long-term field data. Encapsulation strategies, suppression of ion migration, and interface modification remain fundamental research areas. Scaling from laboratory devices to gigawatt-scale manufacturing poses significant barriers, since uniformity, defect control, and yield usually degrade when transitioning to large-scale modules. Solution-processed and printed technologies must demonstrate throughput and reproducibility comparable to crystalline silicon.

Additionally, supply chain maturity, including precursor purity, solvent recovery, and equipment standardization, is still under development. Bankability is another major challenge, as even high efficiency is not financeable without demonstrated lifetimes of 20–30 years, and possible investors require third-party validation, factory audits, and reliable degradation models. The lack of long-term field data is one of the main limitations for perovskite and tandem technologies. In terms of investment cost, crystalline silicon still dominates due to economies of scale and mature supply chains. Emerging technologies must either outperform silicon in cost, achieve higher efficiencies, or enable novel applications such as flexible devices, Building-Integrated Photovoltaics (BIPV), and indoor PV. Their learning curves are still less developed compared to silicon. Manufacturing capital expenditures (CAPEX) also present challenges.

Technology based on vapor deposition and tandem architecture often needs costly equipment, and retrofitting existing silicon production lines is not straightforward. Early markets, such as IoT, portable electronics, and BIPV, are smaller and less standardized, slowing large-scale adoption. Moreover, grid integration requirements must also be addressed. Inverters, power electronics, and safety systems must comply with strict grid codes. Innovative technologies must integrate seamlessly with existing infrastructure. Furthermore, environmental and regulatory considerations are increasingly important as the presence of lead in perovskites raises concerns, and regulations related to recycling and lifecycle impact are becoming stricter. Life cycle assessment (LCA) and circular economy strategies are now essential components of technology development. Also, policy mechanisms like feed-in tariffs, tax incentives, and green hydrogen subsidies can accelerate industrial adoption. In addition, government-funded pilot plants reduce technological risk, while public–private partnerships help to generate field data, which are indispensable for effective bankability. Some of the strategic routes to overcome these barriers include:

- Designing devices to meet IEC standards and real-world operating conditions.
- Prioritizing encapsulation, interface stability, and defect control.
- Combining scalable deposition techniques (e.g., roll-to-roll, hybrid processes).
- Aligning research efforts with national energy and industrial policies.
- Engaging early with certification bodies and regulators.

Finally, smaller markets like indoor solar, IoT devices, aerospace, portable gadgets, and building-integrated solar are easier to enter because they do not require very high reliability and they adopt new technology more quickly. Table 12 summarizes the main barriers, corresponding issues and strategic responses of the PV and optoelectronic energy technologies.

A bigger obstacle to introducing PV technologies into the market is the mix of bankability, learning curve progress, and regulatory approval, which together decide whether a promising prototype can become a financeable energy product. Concerning bankability, it remains constrained by the limited available long-term field data, since even high efficiency is not financeable without demonstrated lifetimes of 20–30 years, and financiers require validated degradation models, factory audits, and third-party certification. As emphasized in recent reliability studies, accelerated tests such as IEC 61215/61730 [92,261] must be complemented by climate-specific degradation modeling and field stress mapping [262,263,264,265,266,267,268]. For perovskites and tandems, the absence of multi-year outdoor datasets, combined with evolving device configurations, complicates the development of degradation models and increases perceived risk premiums. This creates a loop: without deployment there is no field data, and without field data large-scale deployment remains restricted.

How learning curves behave also affects how likely a technology is to succeed in the market. Crystalline silicon benefits from decades of cumulative capacity growth and learning rates near 20%, which is consistent with Swanson’s Law and supported by empirical datasets and ITRPV projections [269,270]. Emerging technologies, by contrast, remain early in their cost-reduction trajectories. Their manufacturing ecosystems—including precursor supply chains, solvent recovery systems, and equipment standardization—are still immature, limiting opportunities for incremental cost reductions. Furthermore, technologies relying on vapor deposition and complex tandem stacks face slower learning rates due to higher CAPEX and lower throughput, whereas solution-processed and printed PV promise steeper future learning curves if challenges in uniformity, defect control, and reliability can be overcome. Integrating the learning curve analysis with lifecycle and environmental assessments, as proposed in recent LCA frameworks [271], gives a clearer picture of how costs and environmental impacts might evolve with production increases.

Regulatory and policy frameworks act as the gatekeepers that determine whether this learning process can begin at scale. Certification standards such as IEC 61215/61730 [92,261] remain necessary but insufficient, as regulators increasingly require life cycle assessments, recycling routes, and compliance with hazardous-substance directives, especially for perovskites containing lead. Also, environmental learning rates and circular-economy fundamentals are becoming central to regulatory approval and public acceptance [271]. Policy mechanisms, including feed-in tariffs, tax incentives, green-hydrogen subsidies, and government-funded pilot lines, can accelerate early deployment, enabling the accumulation of field data and the initiation of learning curve cost reductions. In contrast, public–private partnerships and early engagement with certification bodies help to align emerging technologies with evolving regulatory expectations, reducing time-to-market and improving bankability.

Together, these factors highlight that commercialization is not only a technical challenge but also a systemic one: emerging PV technologies must simultaneously prove reliability, achieve cost declines through learning, and navigate increasingly stringent regulatory landscapes. Coordination across research, manufacturing, certification, and policy domains is therefore essential for perovskites, tandems, and other next-generation optoelectronic technologies to reach large-scale market viability.

## 9. Conclusions

The trajectory of next-generation optoelectronic and photovoltaic energy systems is defined less by incremental efficiency gains and more by the convergence of materials innovation, scalable manufacturing, long-term reliability, intelligent control, system-level integration, and sustainability. Across emerging technologies, such as perovskites, tandem architectures, quantum-engineered absorbers, and photonic structures, performance continues to advance rapidly, yet efficiency is no longer the primary barrier to deployment. Instead, stability, defect tolerance, and environmental durability have become the central determinants of technological viability.

In contrast, achieving commercial relevance requires manufacturing approaches that are not only high-throughput but also reproducible and compatible with large-area production. Hybrid manufacturing, combining precision vapor-deposited layers with low-cost printed and coated absorbers, offers a promising pathway toward industrial scalability. Nonetheless, accelerated aging studies and outdoor data consistently show that encapsulation quality, interface stability, and resistance to moisture, oxygen, heat, and UV radiation govern device lifetime. Demonstrating durability comparable to crystalline silicon remains essential for bankability and investor confidence.

As photovoltaic and optoelectronic devices evolve, they will increasingly function as components of integrated energy systems rather than standalone energy generators. Coupling generation with storage in batteries, hydrogen, and flow systems, alongside advanced power electronics, sensing networks, and AI-driven optimization will transform intermittent resources into predictable, grid-interactive assets. Digital twins, predictive maintenance, and real-time optimization will become standard features of system operation, enabling high renewable penetration without compromising stability.

Sustainability will act as a hard design constraint. Low-temperature, low-energy manufacturing; reduced reliance on critical raw materials; and circular design strategies enabling repair, disassembly, and recycling will increasingly shape both research priorities and market adoption. Policy frameworks and carbon-aware procurement will favor technologies with low embodied energy and well-defined end-of-life pathways.

Taken together, the field is moving toward optoelectronic energy systems that are material-innovative, manufacturing-scalable, reliability-driven, digitally intelligent, system-integrated, and environmentally responsible. Early commercialization will occur in high-value niches such as BIPV, lightweight and flexible modules, indoor PV, IoT, and aerospace, while broader deployment will follow as reliability and certification frameworks mature. The overarching opportunity is to design technologies that achieve high performance and reshape how energy is generated, stored, transmitted, and used within robust and sustainable energy infrastructures.

## Figures and Tables

**Table 1:** Characteristics, benefits and application of the main optoelectronic materials.

Material	Bandgap Type	Benefits	Applications	References
Silicon	Indirect	Scalable Low-cost manufacturing	PV modules Tandem cells	[24,25,26]
Gallium Arsenide	Direct	High efficiency Radiation tolerance	Space PV Tandem cells	[27,28,29,30]
Indium Phosphide	Direct	High surface chemistry compatibility High carrier mobility	Photodetectors PEC devices	[31,32,33,34,35,36,37]

**Table 2:** Main benefits and challenges associated with perovskites, organic semiconductors, and TMDs.

Material Class	Benefits	Challenges	References
Perovskites	High efficiency Adjsutable bandgap Low-cost manufacturing Strong absorption	Instability Lead toxicity	[38,39,40,41,42,43,44,45,46,47,48,49,50,51]
Organic Semiconductors	Flexibility and lightweight Roll-to-roll fabrication Adjustable chemistry	Lower mobility Degradation under oxygen and light	[52,53,54,55,56,57]
TMDs	Atomic-scale thickness Excitonic effects Mechanical robustness	Scalable growth Control of defects	[58,59,60,61,62,63]

**Table 3:** Fundamental benefits and challenges of the nanostructured materials for optoelectronic devices.

Nanostructure	Benefits	Challenges/Limitations	References
Quantum dots	Size-tunable bandgap Multiple Exciton Generation Solution processing	Surface traps states Toxicity	[65,66,67,68,69,70]
Nanowires	1D charge transport Light trapping Defect tolerance	Growth complexity Alignment control	[71,72,73,74]
Plasmonics	Strong near-field enhancement Hot carriers Light scattering	Metal losses Thermal instability Integration complexity	[75,76,77,78,79]

**Table 4:** Stability, toxicity, and sustainability comparison between traditional semiconductors, emerging materials, and nanostructured materials.

Issue	Traditional Semiconductors	Emerging Materials	Nanostructured Materials
Stability	Excellent long-term durability	Moderate; improving with engineering	Highly variable Surface dependency
Toxicity	Generally low	Can be high (e.g., lead, cadmium)	Depends on composition and size
Sustainability	High energy cost but recyclable	Low energy processing Resource concerns Resource concerns	Requires lifecycle management

**Table 5:** Overview of standardized stability testing protocols for optoelectronic materials and devices.

Protocol	Class of Materials	Stress Conditions	Duration	Addressed Degradation Issues
IEC 61215 [92]	III-IV c-Si	Thermal cycling Damp heat UV exposition	1000 h	Long-term field reliability
ISOS-L-1/2/3 [94]	Perovskites Organics	Continuous and cycled light soaking	100–1000 h	Photochemical and thermal degradation
ISOS-D-1/2/3 [94]	Perovskites Hybrids	Dark storage—ambient, elevated temperature and relative humidity	Weeks-months	Intrinsic and moisture stability
ISOS-T [94]	All classes	Thermal stress (65–85 °C)	500–1000 h	Thermal Decomposition

**Table 6:** Main benefits, limitations and applications of the PV technologies.

PV Type	Benefits	Limitations	Applications
Crystalline silicon	High efficiency Long lifetime	Rigid Energy-intensive processing	Utility-scale Rooftop
Thin-Film	Flexible and lightweight Low-cost	Lower efficiency material scarcity	BIPV portable devices
Tandem	Higher efficiency potential	Stability Integration complexity	Advanced modules
Multijunction	Record efficiencies	Very high cost	Space CPV

**Table 7:** Benefits, efficiency potential, and challenges of perovskite technologies.

Technological Option	Benefits	Efficiency Potential	Challenges
Perovskite Single Junction	Low-cost processing Adjustable bandgap	>26%	Stability Lead toxicity
Perovskite–Silicon Tandem	Highest efficiency Complementary absorption	>30%	Integration complexity
			Long-term durability
Perovskite–Organic Hybrid	Flexible and lightweight Semitransparency ability	~20%	Organic degradation; interface stability
Perovskite–QD Hybrid	Infrared absorption Spectral tunability	>25%	QD toxicity Interface control Stability

**Table 8:** Benefits and limitations of light management strategies for concentrator photovoltaics.

Strategy	Benefits	Limitations
CPV	Very high efficiency under concentrated light Reduced active cell area High performance in high-DNI regions	Requires precise solar tracking High thermal load
ARCs	Reduced reflection losses Increased short-circuit current	Limited spectral bandwidth Degradation over time
Surface texturing	Increased light trapping and absorption	Fabrication complexity Surface recombination
Plasmonics/Photonics	Nanoscale light control Enhanced absorption in ultrathin films	Optical losses Integration challenges
Back Reflectors	Increased optical path length Improved infrared absorption	Parasitic absorption losses
Spectrum Splitting	Optimized spectral utilization High-efficiency tandems systems	Optical alignment complexity Additional cost

**Table 9:** Comparison of degradation mechanisms across PV technologies.

Mechanism	Crystalline Silicon	Thin Films ((CdTe, CIGS)	Perovskites	Organics	Nanostructured Devices
Thermal Degradation	Low	Moderate	High	High	Variable
Moisture Sensitivity	Low	Moderate	Very High	High	High
Ion Migration	None	Low	Very High	Moderate	Material-dependent
UV Instability	Low	Moderate	High	High	Moderate
Mechanical Stress	Moderate	Low-Moderate	Moderate	Low	Low-High

**Table 10:** Characteristics and roles of electrochemical storage mechanisms in solar systems.

Technological Option	Round-trip Efficiency	Energy Density	Power Response	Cycle Life	Roles in Solar Systems
Lithium-ion Batteries	85–95%	High	Fast (milliseconds-to-seconds)	High (3000–10,000)	Daily cycling, short- to medium-term storage, grid services
Sodium-ion and other ion Batteries	80–90%	Medium	Fast	Medium–high	Low-cost stationary storage Large-scale PV plants
Flow Batteries	60–75%	Low–medium (tank-limited)	Fast	Very high (more than 10,000 cycles)	Long-duration storage, high-cycle microgrids
Supercapacitors	>90%	Low	Ultra-fast	Extremely high	Power smoothing Transient buffering Power electronics support
Hydrogen (electrolyzer, storage, and fuel cell)	30–45% system-level	Very high	Slow–medium	High	Seasonal storage Backup power Sector coupling (mobility, industry)

**Table 11:** Benefits, limitations, and applications of the main optoelectronic manufacturing techniques.

Technique	Benefits	Limitations	Applications
Solution Processing	Low cost	Sensitive to environment	Perovskites
	Flexible substrates	Reproducibility issues	Organic semiconductors
	Enhanced scalability		Quantum dots
Vapor Deposition	High purity	Costly	Silicon
	Fine control Stable films	Vacuum-based	III-V TMDs Passivation layers
Printing	High throughput	Ink challenges	Flexible PV
	Additive Flexible	Lower resolution	electrodes Large-area devices

**Table 12:** Main barriers, associated challenges, and strategic responses for emerging photovoltaic and optoelectronic energy technologies.

Barrier Category	Challenges	Strategic Responses
Material Stability and Reliability	Sensitivity to moisture, oxygen, UV radiation, and thermal stress Ion migration Interfacial degradation Limited long-term field data	Advanced encapsulation Compositional engineering Interface passivation Accelerated aging protocols and field validation.
Manufacturing Scale-Up	Loss of uniformity and yield in scaling Defect control Limited reproducibility in solution-processed techniques Immature supply chains	Scalable deposition methods (roll-to-roll, vapor-assisted) Process standardization Improved precursor purity In-line quality control
Bankability and Investor Confidence	Lack of 20–30 year lifetime data Uncertainty in degradation models Limited third party validation	Long-term outdoor testing; certification (IEC standards) Independent performance validation Robust degradation modeling
Cost Competitiveness	Dominance of crystalline silicon due to economies of scale High initial costs Slow learning curves	Material usage reduction Efficiency improvement Niche applications (BIPV, flexible PV, indoor PV)
Manufacturing CAPEX	High cost for advanced deposition systems and tandem architecture Difficulty in retrofitting silicon production lines	Hybrid manufacturing Modular production Leveraging existing infrastructure
Grid Integration	Compliance with grid codes Inverter compatibility Variability and intermittency of solar generation	Advanced power electronics Smart inverters Integration with energy storage systems Grid-aware system design
Environmental and Regulatory Constraints	Toxicity (e.g., lead in perovskites) Stricter recycling regulations Lifecycle impact requirements	Lead-free materials recycling procedures LCA integration Circular economy
Policy and Market Adoption	Uncertainty in regulatory frameworks Dependence on subsidies Slow adoption of new technologies	Feed-in tariffs Tax incentives Green hydrogen policies Public–private partnerships Pilot-scale projects
Early Market Entry	Limited large-scale deployment pathways lack of standardized markets for emerging applications	Niche markets (IoT, BIPV, aerospace, portable devices) Rapid prototyping and deployment cycles

## Data Availability

The data presented in this study is available on request from the corresponding author.

## Abbreviations

The following abbreviations are used in this manuscript:

COF	Covalent Organic Framework
CVD	Chemical Vapor Deposition
MOF	Metal–Organic Framework
PVD	Physical Vapor Deposition
TMD	Transition-Metal Dichalcogenide
MEG	Multiple Exciton Generation
LSPR	Localized Surface Plasmon Resonance
LCA	Life Cycle Assessment
CIGS	Copper Indium Gallium Selenide
CPV	Concentrator Photovoltaics
IBC	Interdigitated Back-Contact
DNI	Direct Normal Irradiance
ARC	Antireflection Coatings
DBR	Distributed Bragg Reflectors
PCE	Power Conversion Efficiency
VOC	Open-Circuit Voltage
JSC	Short-Circuit Current Density
FF	Fill Factor
MPPT	Maximum Power Point Tracking
PEC	Photoelectrochemical
HER	Hydrogen Evolution Reaction
OER	Oxygen Evolution Reaction
STH	Solar-to-Hydrogen
ALD	Atomic Layer Deposition
STF	Solar-to-Fuel Efficiency
TOF	Turnover Frequency
BESS	Battery Energy Storage System
EMS	Energy Management System
SoC	State of Charge
FBG	Fiber Bragg Grafting
OCT	Optical Current Transformer
OVS	Optical Voltage Sensors
DFOS	Distributed Fiber-Optic Sensor
PIC	Photonic Integrated Circuit
MMC	Modular Multilevel Converter
DER	Distributed Energy Resource
FACTS	Flexible AC Transmission System
PON	Passive Optical Network
DH	Damp Heat
TC	Thermal Cycling
LID	Light-Induced Degradation
LETID	Light and Elevated Temperature Induced Degradation
LCCA	Life-Cycle Cost Analysis
SQ	Shockley–Queisser
SRH	Shockley–Read–Hall
DFT	Density-Functional-Theory
FDTD	Finite-Difference Time-Domain
PINN	Physics-Informed Neural Network
CNN	Convolutional Neural Network
VPP	Virtual Power Plant
OPV	Organic Photovoltaics
